# Outcome measures used in adolescent sport-related concussion research: a scoping review

**DOI:** 10.1136/bmjopen-2023-075590

**Published:** 2024-09-10

**Authors:** Connor Shane McKee, Chris Bleakley, Alan Rankin, Mark Matthews

**Affiliations:** 1 School of Health Sciences, Ulster University, Belfast, Northern Ireland; 2 Sports Medicine NI, Belfast, UK; 3 School of Sport, Ulster University, Belfast, Northern Ireland

**Keywords:** Adolescent, SPORTS MEDICINE, Prognosis, Paediatric neurology

## Abstract

**ABSTRACT:**

**Objectives:**

To provide an overview of the outcome measures currently used after sports-related concussion (SRC) in adolescents, categorising by the constructs they assess, follow-up duration and their feasibility of use.

**Design:**

Scoping review.

**Data sources:**

We searched three electronic databases (MEDLINE, EMBASE and CINAHL). We also undertook citation tracking of the included articles and searched for ongoing or unpublished trials using ClinicalTrials.gov and Theses Global.

**Eligibility criteria:**

Studies tracking concussion recovery in adolescent athletes.

**Results:**

15 782 records were identified. After initial title and abstract screening, we retrieved 87 studies for full-text screening, with 75 studies fulfilling the eligibility criteria and included in the review, comprising 13 107 participants (9480 male, 3615 female and 12 unreported), ranging in age from 5 to 19 years. 46 different outcome measures were used, with Post-Concussion Symptom Scale (n=42) and Immediate Post-Concussion Assessment and Cognitive Testing (n=21) the most common. Most outcome measures quantified aspects of sensorimotor function including balance, oculomotor function and cognition. Follow-up duration ranged from 7 days to 1 year. 60% of studies ceased follow-up assessments within 6 weeks post-SRC.

**Conclusions:**

Adolescent SRC literature uses a wide range of outcome measures. Most research quantifies cognitive/fatigue domains in the acute/subacute stages post-SRC, using male participants. Other key domains such as anxiety/mood, migraine and key modifiers (cervical and sleep disturbance) are less well represented in the literature. Many of the outcome measures used in current research are associated with high cost and require highly qualified examiners, creating barriers to their implementation in some adolescent sporting environments.

**Study registration:**

https://doi.org/10.17605/OSF.IO/N937E

**Strengths and limitations of this study**

- This scoping review provides an overview of a broad research area, which can help inform future studies in the field of adolescent sport-related concussion research.
- This scoping review followed a prepublished methodology.
- As this was a scoping review, we did not assess the quality of included studies. This means that the findings may be influenced by studies with varying methodological rigour.

## Background

 A sport-related concussion (SRC) is defined as a complex pathophysiological process affecting the brain, induced by biomechanical forces.^1 2^ The fast-paced, competitive nature of sport places young athletes at high risk of concussion. Popular contact and collision sports such as school-age rugby union have the highest incidence of concussion, estimated at 6.01 per 1000 game hours.^3^ Younger athletes are also more likely to have a protracted and unpredictable recovery from concussion, compared with adults.^4^,^6^

The clinical and behavioural manifestations of SRC are highly heterogeneous.^2 7^ In 2014, a clinical model^8^ described six different constructs associated with concussion: (1) cognitive/ fatigue, (2) vestibular, (3) ocular, (4) post-traumatic migraine, (5) anxiety/mood and (6) cervical. In some instances, athletes may present with a single, clearly defined construct, but most will present with more than one, creating a challenge for clinicians who are involved in diagnosis, tracking recovery and informing return to play decisions.^8^,^14^

A battery of tests is currently recommended for both assessing and monitoring recovery in adolescent athletes’ postconcussion.^15 16^ One widely used assessment battery is the Standard Concussion Assessment Tool (SCAT5), which covers several constructs including symptoms, physical signs, balance, behaviour and cognitive impairment.^1^ Although there is consensus that a multidomain assessment is necessary for suspected concussion in adolescent athletes, the optimal combination of methods is unclear.^17 18^ Key symptoms may fluctuate due to adolescent growth and maturation, making it more difficult to create diagnostic cut points or track minimum important changes.^19 20^ Other forms of assessment require expensive equipment and/or access to specialist physicians, which may only be feasible within a professional sporting environment.^1718 20^,^22^

As technology and research develop, it is increasingly difficult for practitioners who work with younger athletes, to select outcome measures which are accurate, cost-effective and feasible in this population. We undertook a scoping review of the current literature for SRC in adolescents. Our primary aims were to determine participant demographics, the outcome tools that are used in a research context, the constructs they assess and the duration of follow-up. As a secondary aim, we examined the resources (time and cost) required to administer each outcome measure and commented on the feasibility of their use within an amateur sporting environment. This review can inform future research in adolescent sport by refining and/or developing key outcomes that are both evidence-based and feasible for assessing and tracking recovery after SRC.

## Methods

### Protocol

This scoping review was conducted by a group of researchers (CSM, MM, AR and CB) using the Preferred Reporting Items for Systematic Reviews and Meta-Analyses Extension for Scoping Reviews (PRISMA-ScR): Checklist and Explanation.^23^ The protocol was developed and published a priori via Open Science Framework (https://osf.io/fz8yt/?view_only=0015ed77afe947808d0d8c901503b323).

### Information sources and literature search

Searches were conducted on three electronic databases (Medline, CINAHL and Embase) from inception to May 2023 using the MeSH term “concussion” combined by ‘AND’ with the secondary MeSH terms “diagnosis” OR “prognosis” OR “assessment” (searched in the title or abstract fields). Study titles and abstracts were imported into Rayyan software, where duplicates were removed. Supplementary searching was undertaken through citation tracking of included articles. Ongoing or unpublished trials were retrieved through ClinicalTrials.gov and Dissertations and Theses Global.

### Eligibility criteria

Eligible studies could have used a case controlled, cohort or prospective design. The study population must have included adolescent athletes aged 13 to 19 years with SRC and at least one follow-up time point. There were no limitations placed on the type of outcome measure or the type of sport, and studies were not limited by published language. Reviews were excluded.

### Data extraction and synthesis

Two reviewers (CSM and MM) independently screened titles and abstracts on Rayyan for relevance, obtaining full-text articles for publications that were potentially relevant. Demographic data (age, sex and sport) were extracted from each study and summarised using counts (%) and means (SD). We determined the number and type of outcome tools used within each study and the construct(s) they assessed. There were six different constructs of interest: cognitive/fatigue, vestibular, ocular, post-traumatic migraine, anxiety/mood and cervical with definitions which were developed from previous research.^24^ An outcome measure was any nondemographic outcome variable that was assessed at least once postconcussion; they could be either subjective (patient-reported outcome measures) or objective (imaging, biomarker and physical performance test). Outcome measures were categorised according to whether they assessed single versus multiple constructs, and survival curves were used to summarise follow-up duration in prospective studies.

### Patient and public involvement

While we recognise the importance of involving patients and the public in research, this scoping review on outcome measures for adolescent SRC did not directly incorporate patient and public involvement.

## Results

**Figure 1:**
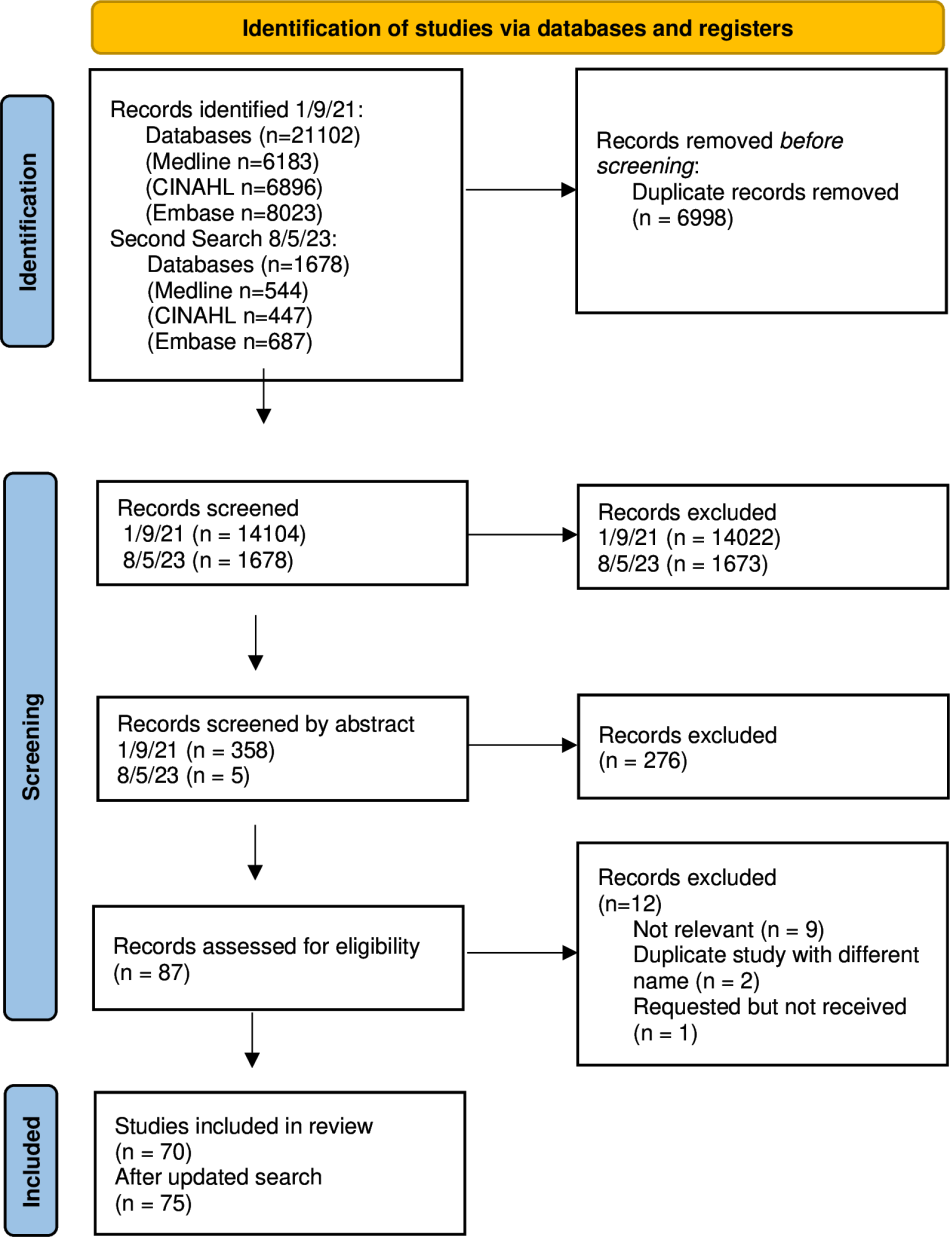
Eligibility flow chart.

The literature search yielded a total of 22 780 citations. 6998 records were removed as duplicates, and 15 782 were removed for not meeting the inclusion criteria. After initial title and abstract screening, 87 studies were included for full-text screening, with 75 studies fulfilling the eligibility criteria and included in the review (figure 1).

### Study characteristics

Study characteristics are presented in online supplemental appendix 1. The included study designs were prospective (n=51), retrospective cohort (n=15), case control (n=6) or other (n=3) (comprised of preliminary reports n=2 and case report n=1). Participants were recruited from across 26 different sports, with most studies focusing exclusively on concussions occurring in American football (n=39 studies) and soccer (n=33 studies). Five studies^25^,^29^ included a proportion of nonathletes but were classed as ‘physically active’ and therefore included as part of this review. Participants were primarily adolescent sport players aged between 5 and 19 years (average age range 11–17). Included studies had an aggregate of n=13 107 participants, 72.4% were male (9480/13107) and 27.6% were female (3615/13107), with the remaining 0.2% of subjects (n=12) not reporting this detail. 64 studies were male dominant (in that they comprised a greater number of male vs female participants). 37 (49%) studies included healthy controls, the majority of which were prospective by design (n=32).

### Outcome measures

**Figure 2:**
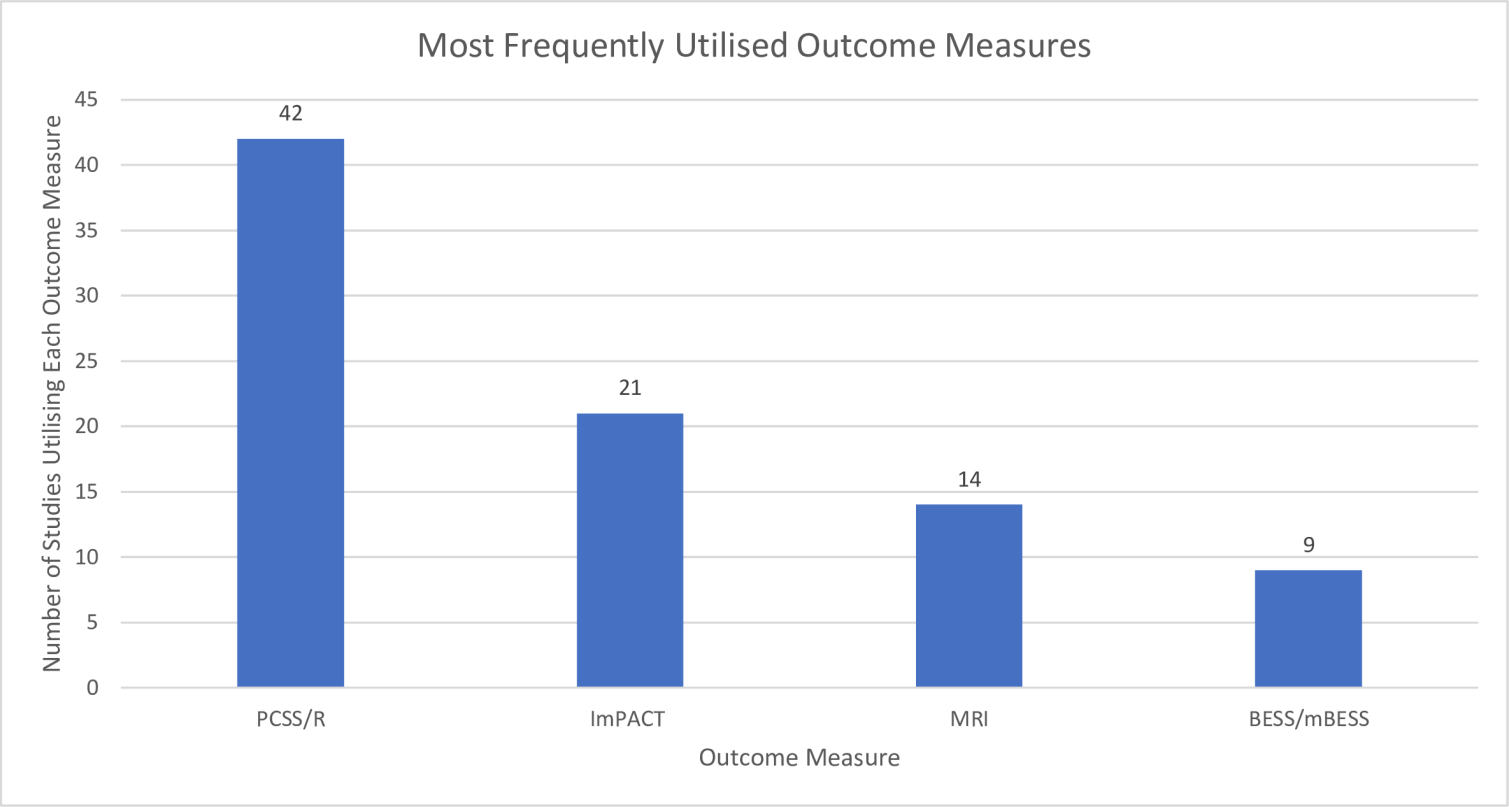
Bar chart of the most common outcome measures in adolescent concussion research. ImPACT, Immediate Post-Concussion Assessment and Cognitive Testing; mBESS, modified Balance Error Scoring System; PCSS/R, Post-Concussion Symptom Scale.

There was an aggregate of 46 different concussion-related outcome measures employed across the included studies. Online supplemental appendix 2 highlights the range of outcome measures included in this review. The Post-Concussion Symptom Scale (PCSS/R) (n=42 studies) and the Immediate Post-Concussion Assessment and Cognitive Testing (ImPACT) test (n=21 studies) were used the most frequently overall and at all stages of longitudinal tracking of recovery (figure 2). MRI (n=14 studies) and Balance Error Scoring System (BESS) or its modification (mBESS) (n=9 studies) were the next most frequently used (figure 2).

The average number of outcome measures used in individual studies was 2.4 (SD 1.1, range 1 to 9), and most studies (n=49; 65%) quantified more than one construct (average of 3.75 constructs per study, range 1–6). Cognitive/fatigue was the most often aligned with construct in the literature, being measured in n=37 studies.

### Constructs

The outcome measures included in this review are often used by clinicians to determine if a concussed patient’s presentation aligns with a set of constructs/modifiers. Outcome measures generally aimed to assess the presentation of the constructs and modifiers: cognitive/fatigue, ocular, vestibular, anxiety/mood, migraine, sleep and neck. SRC outcome measures using imaging or biomarkers were also represented and included MRI, ECG, electroencephalogram and glial and neuronal blood biomarkers. 19 studies aligned with a single construct or biomarker, most commonly cognitive/fatigue (n=11), vestibular (n=6), anxiety/mood (n=1) and biomarkers (n=1).

**Figure 3:**
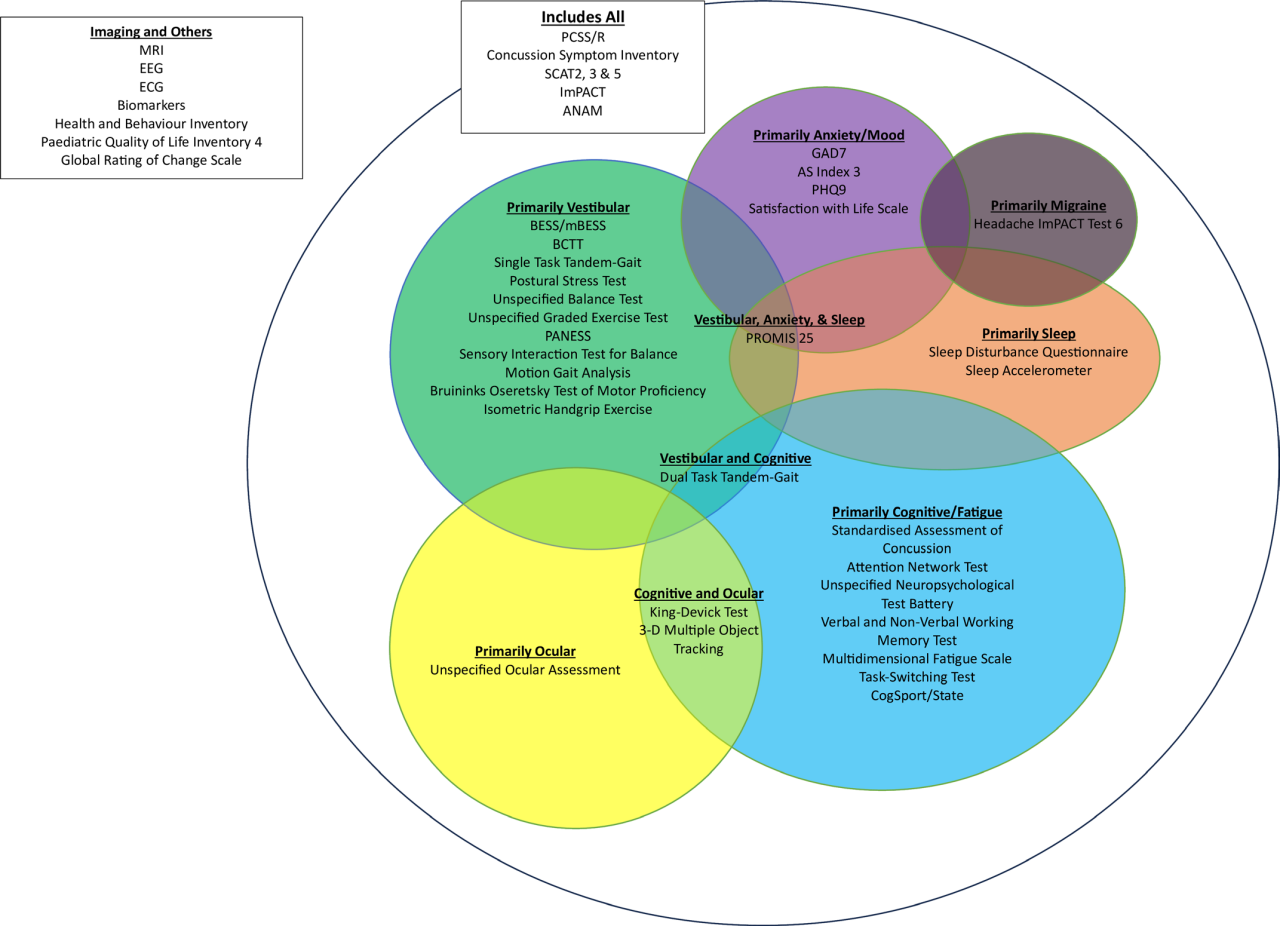
Venn diagram which constructs and modifiers align with the outcome measures. ANAM, Automated Neurophysiological Assessment Metrics; AS, Anxiety Score; BCTT, Buffalo Concussion Treadmill Test; GAD7, Generalised Anxiety Disorder-7; ImPACT, Immediate Post-Concussion Assessment and Cognitive Testing; mBESS, modified Balance Error Scoring System; PANESS, Physical and Neurological Examination for Soft Signs; PCSS/R, Post-Concussion Symptom Scale; PHQ9, Patient Health Questionnaire 9; PROMIS 25, Patient-Reported Outcomes Measurement Information System; SCAT, Standard Concussion Assessment Tool.

Some of the outcome measures that were included (n=10) aimed to align with more than one construct or modifier (figure 3). Several outcome measures including PCSS/R, Concussion Symptom Inventory, SCAT2, SCAT3, SCAT5, ImPACT and the Automated Neurophysiological Assessment Metrics (ANAM) aligned with multiple constructs and modifiers. The majority of outcome measures aimed to align with only one construct or modifier.

### Follow-up assessments

**Figure 4:**
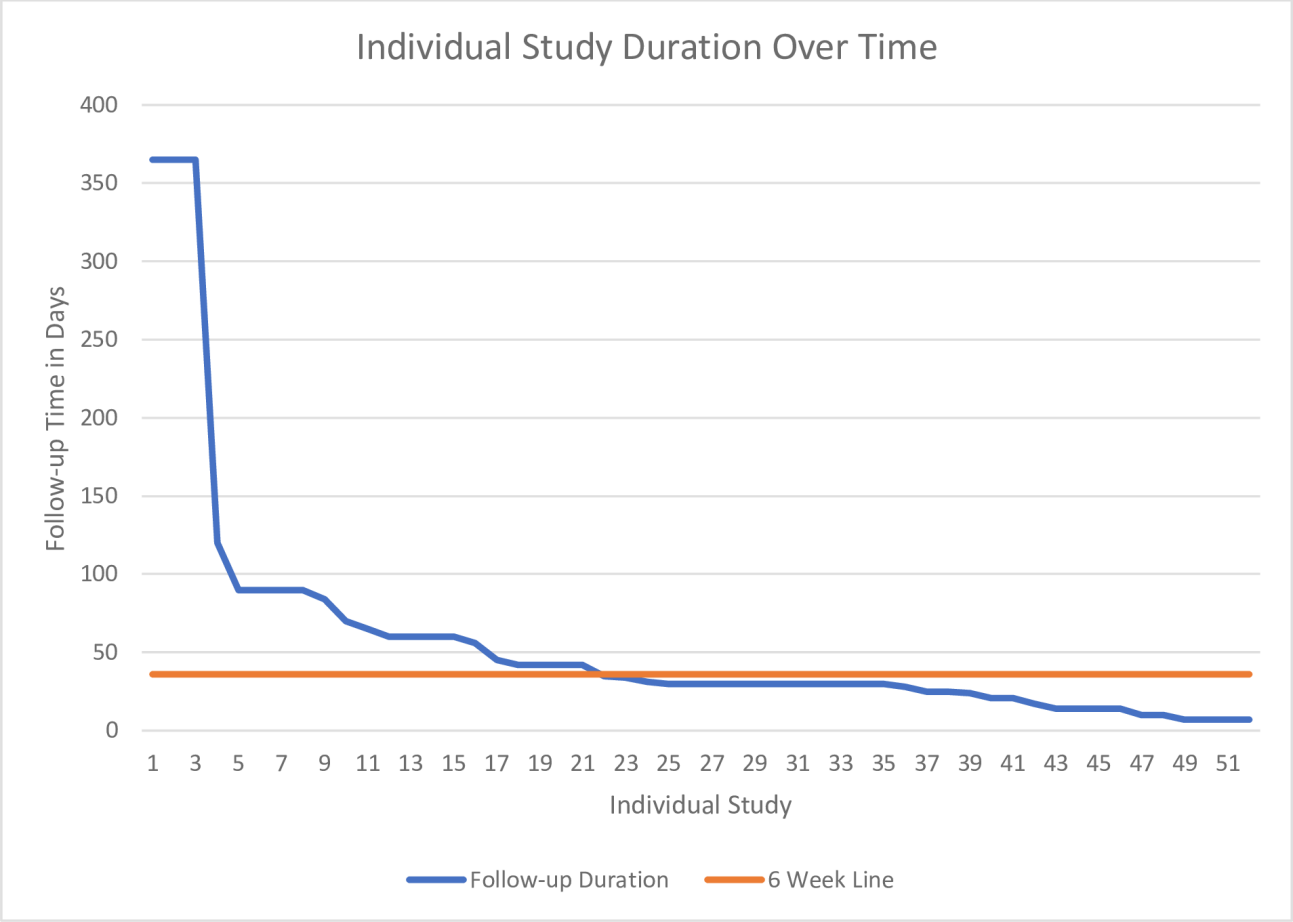
Line graph of the study duration over time.

52 studies provided longitudinal data (including length of the longest follow-up). Figure 4 summarises the length of follow-up time used across studies. The average number of follow-ups undertaken was 3.6 (range 1–9). 25 (48%) of studies completed their first follow-up within 72 hours postconcussion. 18 (35%) completed their first follow-up within 10 days postconcussion. In total, 43 (83%) of studies conducted their first follow-up within 10 days of SRC. By 6 weeks postconcussion, 60% of studies had ceased follow-up assessments, and only three studies continued follow-ups for up to a year (figure 4; orange line).

### Outcome measure feasibility

We determined that 54% (25/46) of outcome measures could be feasibly applied in an adolescent sporting environment (ie, lower cost and without specialist equipment); these were generally questionnaire-based outcome measures and included PCSS/R, Generalised Anxiety Disorder-7 (GAD-7), Health and Behaviour Inventory, BESS/mBESS, Single and Dual-Task Tandem Gait, Post-Concussion Symptom Inventory, Anxiety Sensitivity Index 3, Global Rating of Change Questions, Headache Impact Test 6, Physical and Neurological Examination for Soft Signs (PANESS), Sensory Interaction Test for Balance, Isometric Handgrip Test, Verbal and Non-Verbal Working-Memory Test, Multidimensional Fatigue Scale, Patient Health Questionnaire 9, Patient-Reported Outcomes Measurement Information System (PROMIS-25), Paediatric Quality of Life (QOL) Inventory 4, Satisfaction with Life Scale, Standardised Assessment of Concussion, Task-Switching Test and the Sleep Disturbance Questionnaire.

A further 39% of outcome measures (18/46) (such as ImPACT, King-Devick and SCAT) could also be feasibly applied in certain situations but would require an additional cost or license fee and/or the participation of trained personnel. The remaining 7% (4/46) of outcome measures were deemed to be less feasible for use in an adolescent sporting environment, as they require both specialist equipment and trained medical personnel. These included EEG, MRI, ECG and biomarker data such as glial fibrillary acidic protein (GFAP), ubiquitin C-terminal hydrolase L1 (UCH-L1), saliva samples and serum cortisol levels, among others.

## Discussion

This scoping review of adolescent SRC research captured a wide range of outcome measures and categorised them by construct. N=75 studies were included, with an aggregate of 13 107 participants, ranging in average age from 11 to 17. Almost three-quarters of participants in the current evidence base are male, with most studies recording outcomes in the acute/subacute periods post-SRC. A total of 46 different outcome measures were employed across the included studies. There was a primary focus on quantifying self-reported symptoms post-SRC, with 46% of studies employing ImPACT and/or PCSS (PCSS also forms part of the ImPACT test battery). Anxiety/mood and post-traumatic migraine were the least assessed constructs in the current literature. Many of the outcome measures used in the current research require specialist examiners and/or incur high costs, creating barriers to their implementation in some adolescent sporting environments.

SRC is a heterogeneous injury that requires a multimodal approach to assessment and management.^24^ This is reflected in the adolescent literature where a range of different outcome measures has been used post-SRC. The ImPACT and PCSS were the most frequently used in this study, and this is likely due to their ease of use, the wide variety of areas they assess and relatively low cost. Both these tools are valid and reliable^30 31^ and form part of the testing battery recommended for athletes with SRC, alongside the Standard Assessment of Concussion (SAC) and the SCAT.^32^ Vestibular and ocular constructs were the next most assessed; these were usually quantified using the BESS and King-Devick test, respectively. BESS measures postural stability or balance across six different conditions and has very good test–retest reliability (0.87 to 0.97 intraclass correlations).^33^ BESS also has good diagnostic properties, with a high specificity (0.91) and sensitivity when used in conjunction with the SAC and a graded symptom checklist.^17^ The King-Devick test is a number naming assessment that measures eye movements, attention and language in order to identify deficits associated with concussion. It has acceptable reliability and responsiveness in adolescents, regardless of age or sex.^34^

SRC can acutely affect a range of neuropsychiatric domains, including mood, behaviour, anxiety and depression.^35^ These symptoms may be further implicated by an athlete’s preinjury psychologic health^35^ or other premorbidities such as migraine.^36^ Therefore, it is important to consider pre-existing conditions at baseline to understand the effects this may have on follow-up assessment. There is growing interest in the longer-term neuropsychiatric impact of concussion, particularly in relation to repetitive concussion and/or head impacts, but well-designed prospective trials are lacking. We found that outcome measures exclusively assessing and tracking anxiety/mood and migraine were less well represented in the current adolescent literature. The most used outcome measures for these domains were the Anxiety Sensitivity Index 3, GAD7 and PCSS. Although the PCSS assesses for anxiety in its questions, it fails to provide a score for anxiety separately which limits its usefulness in assessing this domain. These outcome measures have strong psychometric properties, and in adolescents, a person’s negative affect and anxiety sensitivity are not just related to concussion outcomes but also predictive of them.^37^ We also found a dearth of outcome measures examining neck (ie, cervical) pathology and sleep disturbance. Although these are not primary constructs underpinning SRC aetiology, these can be important concussion modifiers^38 39^ and therefore merit further examination in prospective studies.

Most follow-up assessments in this study were undertaken between day 3 and day 21 postconcussion with few extending beyond this subacute period. In a recent review involving 21 966 patients (both adolescent and adult), 80% of prospective studies reported a median time to return to sports post-SRC of 21 days; however, a subset of people experience protracted recoveries from concussion.^40^ Nonelite, younger athletes remain most likely to experience protracted and unpredictable recovery after SRC.^40^ To fully understand the trajectory of recovery in young athletes, follow-ups must be extended further beyond the acute and subacute phases. Prospective data suggest subgroups of adolescents continue to present with impairments to their vision,^26^ heart rate response,^41^ cerebral autoregulation^42^ and anxiety sensitivity^37^ at 6 weeks post-SRC, with others reporting altered cerebral blood flow in paediatric patients for up to 1 year post-SRC, despite full clinical and neurocognitive recovery.^43^

Females remain under-represented in SRC research with almost three-quarters of participants (72%) being male. There is a similar disparity in the adult concussion literature, where only 12.5% of research participants were females^2^; other audits show that there is an under-representation of female participants across the wider sports medicine literature.^44^ Sex is a key biological variable affecting SRC risk and recovery. Prospective epidemiological studies suggest that females are more vulnerable to concussion,^45^ and in data-derived from sex-comparable sports (eg, baseball/softball, basketball, ice hockey and soccer), females have 1.4 times higher overall concussion rates than males.^46^ There is also concern that females may be at greater risk for protracted recovery.

A recent scoping review^47^ involving sporting and military personnel found that many outcome measures lacked clinical feasibility, due to their space/time constraints and costs. In the current review, 47% of outcome tools used in the literature were considered to be difficult or less feasible to employ in an adolescent sporting environment. This was primarily due to their high cost and/or the requirement of a specialist physician or test administrator. Future research in adolescents should seek to employ outcome measures which are more feasible for use in this environment.

Most athletes with SRC present with impairments which align with more than one construct. The minimum standards for returning to competitive sport are full resolution of all postconcussion-related symptoms, normal balance and cognitive function.^16^ Few included studies employed testing batteries that covered all such domains (symptoms, balance and cognitive assessment).^2645 48^,^53^ We also found that most of the physical assessment tasks were static and generally did not include a dual-task element or dynamic stimuli.^41 54^ To better inform clinicians’ return-to-play decision-making, future research must incorporate outcome measures that are more specific to the complex multidimensional needs of the athlete.^16 55^

### Limitations

As this was a scoping review, we included studies with a wide range of methodologies. Although the primary aim of most studies was similar, to prospectively track recovery after SRC (ie, a similar result), the complex aetiology of concussion meant that there was a wide range of outcome measures, many of which assessed multiple constructs. The selection criteria also limited us to studies that included participants with concussion; we acknowledge that some assessment protocols will have been overlooked (as they have only been implemented in healthy participants) but could be valuable in the future. We also acknowledge that generalisation to nonsports mechanisms of injury is limited based on the focus of this scoping review. A further limitation includes the lack of standardised definition of concussion throughout studies.^56^

## Conclusions

This study found that the most frequently assessed outcome measures used in adolescent sports-related concussion research were ImPACT and PCSS. Cognitive/fatigue was the most frequently reported construct, which may reflect the nature of outcome measures and testing methods. Despite the known sex-based differences affecting outcome and recovery, females are under-represented in this field of research, comprising a minority of all study participants. A wide range of outcome measures used in sports-related concussion may be less feasible to implement in an adolescent sporting environment due to associated costs or requirements of highly qualified persons. With evidence indicated a subset of adolescents have a protracted recovery from sports-related concussions, future research needs to incorporate longer follow-up period to explore and quantify possible persistent deficits associated with adolescent sports-related concussion.

## supplementary material

10.1136/bmjopen-2023-075590 online supplemental file 1

10.1136/bmjopen-2023-075590 online supplemental file 2

## Data Availability

Data are available upon reasonable request.
