# Supplementary material for: Outcome measures used in adolescent sport-related concussion research: a scoping review
Source: BMJ Open. 2024 Sep 10;14(9):e075590. doi: 10.1136/bmjopen-2023-075590 (PMC11409241; doi:10.1136/bmjopen-2023-075590)
Supplement: online supplemental file 1 [file bmjopen-14-9-s001.pdf]

Appendix 1- Characteristics of studies that met the inclusion criteria

Study	Participant Characteristics	Sport(s)	Assessment Method(s)	Follow-up
(55)	Age: 13-18<br>Male 72: Female 58<br>Concussed 130: Control 0	Unknown	Buffalo Concussion Treadmill Test	<6 Weeks
(56)	Age: 12-17<br>Male 29: Female 7<br>Concussed 36: Control 0	Ice Hockey	Biomarkers, SCAT3	<6 weeks
(57)	Age: <18<br>Male 47: Female 18<br>Concussed 65: Control 0	Unknown	Unspecified Graded Exercise Test	n/a
(25)	Age: 5-18<br>Male 179: Female 253<br>Concussed 432: Control 0	Unknown	PCSS, Dual- task tandem gait, VOMS	n/a
(40)	Age: 15<br>Male 26: Female 14<br>Concussed 19: Control 16	Ice Hockey and US Football	Isometric handgrip exercise	<3 months
(58)	Age: 8-18<br>Male 210: Female 108<br>Concussed 318: Control 0	Ice Hockey, US Football, Soccer and Basketball	Post-Concussion Symptom Scale	<6 weeks
(59)	Age: 13-19<br>Male 19: Female 7<br>Concussed 13: Control 13	Contact Sports	Brain functional connectivity	<6 weeks
(60)	Age: 12-18<br>Male 55: Female 64<br>Concussed 65: Control 54	Soccer, Hockey, Rugby, Basketball, US Football, Other	ImPACT and PCSS	<3 months
(61)	Age: n/a<br>Male 323: Female 96<br>Concussed 14: Control 405	US Football, Wrestling, Basketball, Soccer, Baseball	ANAM	<10 days

(62)	Age: 13-18<br>Male 20: Female 20<br>Concussed 20: Control 20	Unknown	Self-reported graded symptom checklist and AS Index-3	<6 weeks
(63)	Age: <18<br>Male 51: Female 7: Unreported 12<br>Concussed 70: Control 0	US Football, Basketball, Cheer, Soccer, Wrestling, Volleyball, Unreported	Patient-reported outcomes measurement information system pediatric-25	<3 weeks
(48)	Age: 6-18<br>Male 97: Female 73<br>Concussed 126: Control 58	Unknown	Health and Behaviour Inventory, mBESS, single and dual task tandem gait	<3 months
(47)	Age: 8-18<br>Male 39: Female 28<br>Concussed 36: Control 31	Unknown	PCSS, Dual-Task gait, event related potentials and eye tracking	<3 months
(64)	Age: 14-19<br>Male 93 : Female 14<br>Concussed 30: Control 77	Unknown	Event related potential (auditory oddball task), captured with EEG called processing negativity	<3 months
(65)	Age: Grade 9-12<br>Male 995 : Female 3<br>Concussed 62: Control 1114	US Football	PCSS, Patient health questionnaire 9 and pediatric quality of life inventory 4.0	<1 year
(66)	Age: n/a<br>Male 64 : Female 0<br>Concussed 8: Control 0	US Football	Cogstate, Brain Network Activation, Clinical Reaction Time, SCAT3 self-reported symptoms and Health Behaviour Inventory and Satisfaction with Life Scale	<6 months
(42)	Age: 15-17<br>Male 3 : Female 3<br>Concussed 6: Control 0	Soccer, Ice Hockey, US Football, Cycling	MRI	<1 year
(67)	Age: 13-16<br>Male 67 : Female 33<br>Concussed 100: Control 0	Soccer, US Football, Basketball, Wrestling, Cross Country, Tennis, Ice Hockey, Volleyball,	ImPACT	<6 weeks

		Baseball, Softball, Cheer, Lacrosse, Athletics, Field Hockey, Gymnastics, Unknown		
(68)	Age: 5-17<br>Male 157 : Female 63<br>Concussed 220: Control 0	Unknown	Post-concussion symptom inventory and CogSport	<3 weeks
(69)	Age: 13-18<br>Male 48 : Female 28<br>Concussed 46: Control 20	Unknown	King-Devick and Graded Treadmill test	<3 weeks
(70)	Age: 14-18<br>Male 30: Female 0<br>Concussed 16: Control 12	US Football	Resting state functional MRI, Diffusion tensor imaging and ImPACT	<6 weeks
(26)	Age: 9-18<br>Male 26 : Female 8<br>Concussed 14: Control 20	Unknown	Three-dimensional multiple object tracking	<6 months
(10)	Age: <19<br>Male 238 : Female 161<br>Concussed 399: Control 0	Soccer, Ice Hockey	VOD, NPC, Smooth pursuits, saccades, VOR.	n/a
(24)	Age: 5-18<br>Male 138 : Female 76<br>Concussed 213: Control 0	Unknown	Post-Concussion Symptom Scale	<10 days
(28)	Age: 11-16<br>Male 10 : Female 1<br>Concussed 11: Control 0	Unknown	Post-Concussion Symptom Scale and Balance Assessment	<6 weeks
(27)	Age: 11-16<br>Male 38 : Female 7<br>Concussed 25: Control 20	Unknown	Biomarker and PCSS	<6 weeks
(49)	Age: n/a<br>Male 217 : Female 77<br>Concussed 105: Control 0	US Football, Basketball, Soccer, Cheer, Other	SCAT-2 and Balance Testing	n/a
(37)	Age: 11-18<br>Male 238 : Female 123	US Football, Wrestling, Lacrosse,	ImPACT	n/a

	Concussed 361: Control 0	Soccer, Basketball, Ice Hockey, Volleyball, Field Hockey, Cheer, Other		
(71)	Age: <19<br>Male 63 : Female 38<br>Concussed 77: Control 0	Baseball, Basketball, Skating, US Football, Ice Hockey, Ringette, Snow Sports, Soccer, Volleyball, Other	PCSS and Ocular Examination	<3 months
(72)	Age: 14-17<br>Male 34 : Female 4<br>Concussed 19: Control 19	US Football, Soccer, Volleyball	Motion gait analysis while simultaneously performing a cognitive task.	<3 months
(73)	Age: n/a<br>Male 20 : Female 3<br>Concussed 23: Control 23	US Football, Soccer, Wrestling, Volleyball	Dual-task walking protocol	<3 months
(74)	Age: 12-19<br>Male 17 : Female 18<br>Concussed 35: Control 0	Basketball, Cheer, US Football, Gymnastics, Soccer, Wrestling, Other	King-Devick, PCSS, ImPACT	<6 months
(75)	Age: 13-18<br>Male 1088 : Female 324<br>Concussed 1412: Control 0	US Football, Soccer, Basketball, Wrestling, Baseball, Softball, Volleyball	No specific diagnostic criteria	<3 months
(50)	Age: 16-17<br>Male 507 : Female 63<br>Concussed 570: Control 166	US Football, Soccer, Lacrosse, Ice Hockey	Graded Symptom Checklist, BESS, SAC and a brief neuropsychological test battery	<3 months
(76)	Age: 11-17<br>Male 18 : Female 6<br>Concussed 12: Control 12	Soccer, US Football, Wrestling	ImPACT, MRI, DTI	<6 weeks
(77)	Age: 14<br>Male 0 : Female 2<br>Concussed 2: Control 0	Field Hockey	ImPACT, Attention Network Test	<6 weeks
(78)	Age: n/a<br>Male 58 : Female 14<br>Concussed 72: Control 2000	Baseball, Basketball, Cheer, US Football, Gymnastics, Ice Hockey, Soccer, Softball, Volleyball, Wrestling	ImPACT	<6 weeks

(79)	Age: 13-18<br>Male 76 : Female 12<br>CC 64: CT 24	US Football, Basketball, Soccer and Other	ImPACT	<10 days
(80)	Age: 12-18<br>Male 15 : Female 7<br>Concussed 10: Control 12	Running, Volleyball, US Football, Baseball, Wrestling, Swimming, Skateboard, Soccer, Basketball	DTI and MRI	<3 months
(81)	Age: n/a<br>Male 108 : Female 0<br>Concussed 108: Control 0	US Football	PCSS and ImPACT	<3 weeks
(60)	Age: 11-18<br>Male 86 : Female 23<br>Concussed 109: Control 0	US Football, Basketball, Soccer, Baseball, Wrestling, Other	PCSS and ImPACT	<3 weeks
(47)	Age: 8-18<br>Male 25 : Female 25<br>Concussed 23: Control 27	Unknown	Single/dual task tandem gait and PCSS	<3 weeks
(82)	Age: 14-19<br>Male 37 : Female 17<br>Concussed 27: Control 27	Unknown	Buffalo Concussion Treadmill Test	<3 weeks
(83)	Age: 13-17<br>Male 23 : Female 12<br>Concussed 15: Control 20	Unknown	Physical and Neurological Examination of Subtle Signs	<6 months
(84)	Age: 11-12<br>Male 13 : Female 1<br>Concussed 14: Control 0	Ice Hockey	Physical and Neurological Examination of Subtle Signs	<6 weeks
(85)	Age: 12-16<br>Male 103 : Female 36<br>Concussed 139: Control 0	Australian Rules Football, Rugby, Other, Fall during play, Riding, Cycling, Equestrian, Skating, Other	VOMS, M-BESS, ImPACT and a concussion symptom-limited graded exercise test.	<6 weeks
(86)	Age: 13-18<br>Male 52 : Female 47<br>Concussed 99: Control 0	Unknown	King-Devick, BCTT and PCSS	<6 weeks

(87)	Age: 14-18<br>Male 44 : Female 19<br>Concussed 63: Control 0	US Football, Basketball, Cheer, Soccer, Wrestling	VOMS and NPC	<3 weeks
(41)	Age: 12-18<br>Male 15 : Female 22<br>Concussed 19: Control 18	Unknown	MRI, SCAT-3 and GAD-7	<3 months
(88)	Age: n/a<br>Male 94 : Female 0<br>Concussed 94: Control 0	US Football	Global rating questions, Pediatric quality of life inventory, multidimensional fatigue scale, and headache impact test 6	<6 weeks
(51)	Age: 10-17<br>Male 1242 : Female 598<br>Concussed 1733: Control 0	US Football, Soccer, Basketball, Wrestling, Lacrosse, Hockey, Volleyball, Cheer, Baseball, Softball, Other	Patient survey symptom score, BESS, SAC, Cogstate	n/a
(89)	Age: n/a<br>Male 481 : Female 433<br>Concussed 77: Control 77	US Football, Cheer, Soccer, lacrosse, Basketball, Wrestling, Volleyball, Field Hockey, other	ImPACT	<3 months
(90)	Age: n/a<br>Male 30 : Female 20<br>Concussed 25: Control 25	Ice Hockey, Basketball, Soccer	ClinicaVR: Classroom-CPT and the VIGIL-CPT	<1 year
(91)	Age: <19<br>Male 235 : Female 116<br>Concussed 351: Control 0	US Football, Basketball, Soccer, Lacrosse, Basketball, Ice Hockey, baseball, Volleyball	Health Behavior inventory, headache severity rating, sleep disturbance questionnaire, BESS, Romberg, tandem gait, ocular assessment	<1 year
(52)	Age: n/a<br>Male 90 : Female 0<br>Concussed 59: Control 31	US Football	Concussion symptom inventory, SAC, BESS, ANAM, EEG	<6 weeks
(5)	Age: 5-18<br>Male 143 : Female 104<br>Concussed 247: Control 0	Unknown	ImPACT, VOMS	Follow-up: n/a
(44)	Age: 13-19<br>Male 110 : Female 37<br>Concussed 147: Control 0	US Football, Soccer, Ice Hockey, Lacrosse, Softball, Basketball	SCAT2, ANAM or ImPACT, and BCTT	<6 weeks

(92)	Age: 8-17<br>Male 144 : Female 54<br>Concussed 198: Control 0	Basketball, Canadian Football, Soccer, Rugby, Ice Hockey	SCAT2	n/a
(93)	Age: 12-19<br>Male 51 : Female 18 : Unreported 12<br>Concussed 95: Control 0	US Football, Soccer, Ice Hockey, Volleyball, Field Hockey, Rugby, Basketball, Wrestling	ImpACT, PCSS	<6 weeks
(94)	Age: 13-18<br>Male 35 : Female 8<br>Concussed 43: Control 0	US Football, Soccer, Basketball, Ice Hockey, Lacrosse, Baseball, Softball	ImPACT	<10 days
(95)	Age: 10-17<br>Male 149 : Female 56 : Unreported 1<br>Concussed 206: Control 15	Hockey, Soccer, lacrosse, Basketball, Baseball, US Football, Other	ImPACT and an Ocular assessment	<1 year
(96)	Age: 14-18<br>Male 36 : Female 4<br>Concussed 20: Control 20	US Football, Soccer, Volleyball, Wrestling, Basketball	Dual-task walking	<3 months
(97)	Age: 13-18<br>Male 18 : Female 14<br>Concussed 32: Control 0	Ice Hockey	ImPACT, PCSS	<3 months
(98)	Age: n/a<br>Male 36 : Female 4<br>Concussed 20: Control 20	US Football, Soccer, Volleyball, Wrestling, Basketball	Attentional Network Test	<3 months
(99)	Age: 11-14<br>Male 17 : Female 0<br>Concussed 17: Control 26	Ice Hockey	DTI, MRI	<3 months
(100)	Age: 10-14<br>Male 17 : Female 4<br>Concussed 21: Control 0	Ice Hockey	Verbal and non-verbal working memory task, PCS-R	<3 months
(11)	Age: 13-19<br>Male 108 : Female 0 : U69<br>Concussed 177: Control 0	US Football	ImPACT	<3 months
(101)	Age: n/a<br>Male 12 : Female 28<br>Concussed 20: Control 20	Basketball, Cheer, US Football, Ice Hockey, Soccer, Softball, Volleyball, Other	Imaging and PCSS	<3 months

(102)	Age: 13-19<br>Male 138 : Female 0<br>Concussed 138: Control 0	US Football	ImPACT	<3 weeks
(53)	Age: 11<br>Male 0 : Female 1<br>Concussed 1: Control 0	Snowboarding	Balance subtest of Bruininks-Oseretsky Test of Motor Proficiency, the pediatric clinical test of sensory interaction for balance, the postural stress test	<3 months
(1)	Age: 15-19<br>Male 131 : Female 0<br>Concussed 16: Control 115	Rugby Union	SCAT3, Cogstate, King-Devick Test	<6 weeks
(2)	Age: 13.83 (±2.02)<br>Male 17 : Female 23<br>Concussed 40 : Control 0	Soccer, American Football	BESS correlated with center-of-pressure (COP) measures.	<6 weeks
(3)	Age: 13-18<br>Male 80 : Female 45<br>Concussed 125: Control 2035	US Football, Basketball, Volleyball, Soccer	PHQ9, SCAT5.	<1 year
(4)	Age: 12-19<br>Male 9 : Female 8<br>Concussed 17 : Control 0	Unknown	Sleep Movement via Accelerometer, PCSS	<6 weeks
(5)	Age: 15-19<br>Male 135 : Female 0<br>Concussed 15 : Control 120	Rugby Union	M-BESS, MRI	<1 Week

1. Cosgrave C, Fuller C, Kung S, Cosgrave M, McFadden C, FranklynMiller A. A comparison of clinical assessment with common diagnostic tools for monitoring concussion recovery in adolescent rugby union players. Physical Therapy in Sport. 2023;61:165-71.
2. Ulman S, Erdman AL, Loewen A, Worrall HM, Tulchin-Francis K, Jones JC, et al. Improvement in balance from diagnosis to return-to-play initiation following a sport-related concussion: BESS scores vs center-of-pressure measures. Brain Injury. 2022;36(8):921-30.
3. Hammer E, Hetzel S, Pfaller A, McGuine T. Longitudinal Assessment of Depressive Symptoms After Sport-Related Concussion in a Cohort of High School Athletes. Sports health. 2021;13(1):31-6.
4. Trbovich AM, Howie EK, Elbin RJ, Ernst N, Stephenson K, Collins MW, et al. The relationship between accelerometer-measured sleep and next day ecological momentary assessment symptom report during sport-related concussion recovery. Sleep Health. 2021;7(4):519-25.
5. Daniels KAJ, Henderson G, Strike S, Cosgrave C, Fuller C, Falvey E. The use of continuous spectral analysis for the assessment of postural stability changes after sports-related concussion. Journal of Biomechanics. 2019;97:109400.

References

1. Echemendia RJ, Meeuwisse W, McCrory P, Davis GA, Putukian M, Leddy J, et al. The Sport Concussion Assessment Tool 5th Edition (SCAT5): Background and rationale. British journal of sports medicine. 2017;51(11):848-50.
2. Moody JR, Feiss RS, Pangelinan MM. A systematic review of acute concussion assessment selection in research. Brain injury. 2019;33(8):967-73.
3. Archbold HA, Rankin AT, Webb M, Nicholas R, Eames NW, Wilson RK, et al. RISUS study: Rugby Injury Surveillance in Ulster Schools. British journal of sports medicine. 2017;51(7):600-6.
4. Alexander DG, Shuttleworth-Edwards AB, Kidd M, Malcolm CM. Mild traumatic brain injuries in early adolescent rugby players: Long-term neurocognitive and academic outcomes. Brain injury. 2015;29(9):1113-25.
5. Corwin DJMD, Wiebe DJP, Zonfrillo MRMDM, Grady MFMD, Robinson RLMSNC, Goodman AMMD, et al. Vestibular Deficits following Youth Concussion. The Journal of pediatrics. 2015;166(5):1221-5.
6. Manzanero S, Elkington LJ, Praet SF, Lovell G, Waddington G, Hughes DC. Post-concussion recovery in children and adolescents: A narrative review. Journal of Concussion. 2017;1:2059700217726874.
7. Collins MW, Kontos AP, Reynolds E, Murawski CD, Fu FH. A comprehensive, targeted approach to the clinical care of athletes following sport-related concussion. Knee surgery, sports traumatology, arthroscopy : official journal of the ESSKA. 2014;22(2):235-46.
8. Yeates KO. Mild traumatic brain injury and postconcussive symptoms in children and adolescents. Journal of the International Neuropsychological Society : JINS. 2010;16(6):953-60.
9. Hides JA, Franettovich Smith MM, Mendis MD, Smith NA, Cooper AJ, Treleaven J, et al. A prospective investigation of changes in the sensorimotor system following sports concussion. An exploratory study. Musculoskeletal science & practice. 2017;29:7-19.
10. Ellis MJ, Cordingley DM, Vis S, Reimer KM, Leiter J, Russell K. Clinical predictors of vestibulo-ocular dysfunction in pediatric sports-related concussion. Journal of Neurosurgery Publishing Group (JNSPG); 2017. p. 38.
11. Lau B, Lovell M, Collins M, Pardini J. Neurocognitive and Symptom Predictors of Recovery in High School Athletes. Clinical journal of sport medicine. 2009;19(3):216-21.
12. Feddermann-Demont N, Echemendia RJ, Schneider KJ, Solomon GS, Hayden KA, Turner M, et al. What domains of clinical function should be assessed after sport-related concussion? A systematic review. British journal of sports medicine. 2017;51(11):903-18.
13. Leung FT, Mendis MD, Franettovich Smith MM, Rahmann A, Treleaven J, Hides JA. Sensorimotor system changes in adolescent rugby players post-concussion: A prospective investigation from the subacute period through to return-to-sport. Musculoskeletal science & practice. 2022;57:102492.
14. Harmon KG, Clugston JR, Dec K, Hainline B, Herring S, Kane SF, et al. American Medical Society for Sports Medicine position statement on concussion in sport. British journal of sports medicine. 2019;53(4):213-25.
15. McCrory P, Meeuwisse W, Dvořák J, Aubry M, Bailes J, Broglio S, et al. Consensus statement on concussion in sport-the 5(th) international conference on concussion in sport held in Berlin, October 2016. British journal of sports medicine. 2017;51(11):838-47.
16. Giza CC, Kutcher JS, Ashwal S, Barth J, Getchius TS, Gioia GA, et al. Summary of evidence-based guideline update: evaluation and management of concussion in sports: report of the Guideline Development Subcommittee of the American Academy of Neurology. Neurology. 2013;80(24):2250-7.
17. Sherry NS, Fazio-Sumrok V, Sufrinko A, Collins MW, Kontos AP. Multimodal Assessment of Sport-Related Concussion. Clinical journal of sport medicine : official journal of the Canadian Academy of Sport Medicine. 2021;31(3):244-9.
18. Halstead ME, Walter KD, Moffatt K, Council On Sports M, Fitness. Sport-Related Concussion in Children and Adolescents. Pediatrics. 2018;142(6):e20183074. doi: 10.1542/peds.2018-3074. Epub 2018 Nov 12.
19. Davis GA, Anderson V, Babl FE, Gioia GA, Giza CC, Meehan W, et al. What is the difference in concussion management in children as compared with adults? A systematic review. British journal of sports medicine. 2017;51(12):949-57.
20. Valovich McLeod TC, Barr WB, McCrea M, Guskiewicz KM. Psychometric and measurement properties of concussion assessment tools in youth sports. Journal of athletic training. 2006;41(4):399-408.
21. McCrea M, Hammeke T, Olsen G, Leo P, Guskiewicz K. Unreported concussion in high school football

players: implications for prevention. Clinical journal of sport medicine : official journal of the Canadian Academy of Sport Medicine. 2004;14(1):13-7.
22. Tricco AC, Lillie E, Zarin W, O'Brien KK, Colquhoun H, Levac D, et al. PRISMA Extension for Scoping Reviews (PRISMA-ScR): Checklist and Explanation. Annals of Internal Medicine. 2018;169(7):467-73.
23. Kontos A, Trbovich A, Sandel N, Emami K, Collins M. Sport-related Concussion Clinical Profiles: Clinical Characteristics, Targeted Treatments, and Preliminary Evidence. Current Sports Medicine Reports. 2019;18:82-92.
24. Corbin-Berrigan L-A, Gagnon I. Postconcussion Symptoms as a Marker of Delayed Recovery in Children and Youth Who Recently Sustained a Concussion: A Brief Report. Clinical journal of sport medicine. 2017;27(3):325-7.
25. Master C, Master S, Wiebe D, Storey E, Lockyer J, Podolak O, et al. Vision and Vestibular System Dysfunction Predicts Prolonged Concussion Recovery in Children. Clinical journal of sport medicine. 2018;28(2):139-45.
26. Corbin-Berrigan L-A, Kowalski K, Faubert J, Christie B, Gagnon I. Three-dimensional multiple object tracking in the pediatric population: the NeuroTracker and its promising role in the management of mild traumatic brain injury. Neuroreport. 2018;29(7):559-63.
27. Rhine T, Babcock L, Zhang N, Leach J, Wade SL. Are UCH-L1 and GFAP promising biomarkers for children with mild traumatic brain injury? Brain injury. 2016;30(10):1231-8.
28. Rhine T, Quatman-Yates C, Clark RA. A longitudinal examination of postural impairments in children with mild traumatic brain injury: implications for acute testing. The journal of head trauma rehabilitation. 2017;32(2):E18-E23.
29. Lovell MR, Iverson GL, Collins MW, Podell K, Johnston KM, Pardini D, et al. Measurement of symptoms following sports-related concussion: reliability and normative data for the post-concussion scale. Applied Neuropsychology. 2006;13(3):166-74.
30. Iverson GL, Lovell MR, Collins MW. Validity of ImPACT for measuring processing speed following sports-related concussion. Journal of clinical and experimental neuropsychology. 2005;27(6):683-9.
31. Kontos AP, Collins MW. Concussion; A Clinical Profile Approach to Assessment and Treatment: American Psychological Association; 2018 2022/12.
32. Riemann BL, Guskiewicz KM, Shields EW. Relationship between Clinical and Forceplate Measures of Postural Stability. Journal of Sport Rehabilitation. 1999;8(2):71-82.
33. Oberlander TJ, Olson BL, Weidauer L. Test-Retest Reliability of the King-Devick Test in an Adolescent Population. Journal of athletic training. 2017;52(5):439-45.
34. Barela M, Wong A, Chamberlain R. Concussion and Psychological Effects: A Review of Recent Literature. Current sports medicine reports. 2023;22(1):24-8.
35. McAllister TW, Wall R. Chapter 16 - Neuropsychiatry of sport-related concussion. In: Hainline B, Stern RA, editors. Handbook of Clinical Neurology. 158: Elsevier; 2018. p. 153-62.
36. Caze T, Williams K, Boucher S, Price A, Abt J, Burkhart S. Influence of Anxiety Sensitivity and Negative Affect on Concussion Outcomes. Orthopaedic Journal of Sports Medicine. 2022;10(5):2325967121S00424.
37. Bock S, Grim R, Barron TF, Wagenheim A, Hu YE, Hendell M, et al. Factors associated with delayed recovery in athletes with concussion treated at a pediatric neurology concussion clinic. Child's nervous system. 2015;31(11):2111-6.
38. Brooks BL, Sayers PQ, Virani S, Rajaram AA, Tomfohr-Madsen L. Insomnia in Adolescents with Slow Recovery from Concussion. Journal of neurotrauma. 2019;36(16):2391-9.
39. Wait TJ, Eck AG, Loose T, Drumm A, Kolaczko JG, Stevanovic O, et al. Median Time to Return to Sports After Concussion Is Within 21 Days in 80% of Published Studies. Arthroscopy : The Journal of Arthroscopic & Related Surgery : Official Publication of the Arthroscopy Association of North America and the International Arthroscopy Association. 2023;39(3):887-901.
40. Woehrle E, Harriss A, Abbott K, Moir M, Balestrini C, Fischer L, et al. Concussion in Adolescents Impairs Heart Rate Response to Brief Handgrip Exercise. Clinical journal of sport medicine. 2020;30(5):e130-e3.
41. Moir M, Balestrini C, Abbott K, Klassen S, Fischer L, Fraser D, et al. An Investigation of Dynamic Cerebral Autoregulation in Adolescent Concussion. Medicine and science in sports and exercise. 2018;50(11):2192-9.
42. Mutch WAC, Ellis MJ, Ryner LN, Morissette MP, Pries PJ, Dufault B, et al. Longitudinal Brain Magnetic Resonance Imaging CO2 Stress Testing in Individual Adolescent Sports-Related Concussion Patients: A Pilot Study. Frontiers in neurology. 2016;7:107.
43. Costello JT, Bieuzen F, Bleakley CM. Where are all the female participants in Sports and Exercise Medicine research? null. 2014;14(8):847-51.

44. Baker JG, Leddy JJ, Darling SR, Shucard J, Makdissi M, Willer BS. Gender Differences in Recovery From Sports-Related Concussion in Adolescents. Clinical pediatrics. 2016;55(8):771-5.
45. Covassin T, Moran R, Elbin RJ. Sex Differences in Reported Concussion Injury Rates and Time Loss From Participation: An Update of the National Collegiate Athletic Association Injury Surveillance Program From 2004-2005 Through 2008-2009. Journal of athletic training. 2016;51(3):189-94.
46. DuPlessis D, Lam E, Xie L, Reed N, Wright FV, Biddiss E, et al. Multi-domain assessment of sports-related and military concussion recovery: A scoping review. (1873-1600 (Electronic)).
47. Howell D, Myer G, Brilliant A, Barber Foss K, Meehan W. Quantitative Multimodal Assessment of Concussion Recovery in Youth Athletes. Clinical journal of sport medicine. 2019;31(2):133-8.
48. Van Deventer KA, Seehusen CN, Walker GA, Wilson JC, Howell DR. The diagnostic and prognostic utility of the dual-task tandem gait test for pediatric concussion. Journal of sport and health science. 2021;10(2):131-7.
49. Miller JH, Gill C, Kuhn EN, Rocque BG, Menendez JY, Neill JA, et al. Predictors of delayed recovery following pediatric sports-related concussion: a case-control study. Journal of neurosurgery Pediatrics. 2016;17(4):491-6.
50. McCrea M, Guskiewicz, Kevin, Randolph C, B. W, Batt, et al. Incidence, Clinical Course, and Predictors of Prolonged Recovery Time Following Sport-Related Concussion in High School and College Athletes. 2013. p. 22.
51. Thomas D, Coxe K, Li H, Pommering T, Young J, Smith G, et al. Length of Recovery From Sports-Related Concussions in Pediatric Patients Treated at Concussion Clinics. Clinical journal of sport medicine. 2018;28(1):56-63.
52. Barr WB, Prichep LS, Chabot R, Powell MR, McCrea M. Measuring brain electrical activity to track recovery from sport-related concussion. Brain injury. 2012;26(1):58-66.
53. Gagnon I, Friedman D, Swaine B, Forget R. Balance Findings in a Child before and after a Mild Head Injury.
54. Fino PC, Parrington L, Pitt W, Martini DN, Chesnutt JC, Chou LS, et al. Detecting gait abnormalities after concussion or mild traumatic brain injury: A systematic review of single-task, dual-task, and complex gait. (1879-2219 (Electronic)).
55. Haider MN, Leddy JJ, Wilber CG, Viera KB, Bezherano I, Wilkins KJ, et al. The Predictive Capacity of the Buffalo Concussion Treadmill Test After Sport-Related Concussion in Adolescents. Frontiers Media SA; 2019.
56. Seeger TA, Tabor J, Sick S, Schneider KJ, Jenne C, La P, et al. The Association of Saliva Cytokines and Pediatric Sports-Related Concussion Outcomes. The journal of head trauma rehabilitation. 2020;35(5):354-62.
57. Popovich M, Sas A, Almeida AA, Freeman J, Alsalaheen B, Lorincz M, et al. Symptom Provocation During Aerobic and Dynamic Supervised Exercise Challenges in Adolescents With Sport-Related Concussion. Journal of athletic training. 2021;56(2):148-56.
58. Howell DR, Brien MJ, Beasley MA, Mannix RC, Meehan Iii WP. Initial somatic symptoms are associated with prolonged symptom duration following concussion in adolescents. Acta Paediatrica. 2016;105(9):e426-e32.
59. Newsome MR, Li X, Lin X, Wilde EA, Ott S, Biekman B, et al. Functional Connectivity Is Altered in Concussed Adolescent Athletes Despite Medical Clearance to Return to Play: A Preliminary Report. Frontiers in neurology. 2016;7:116.
60. Hang B, Babcock L, Hornung R, Ho M, Pomerantz W. Can Computerized Neuropsychological Testing in the Emergency Department Predict Recovery for Young Athletes With Concussions? Pediatric emergency care. 2015;31(10):688-93.
61. Sim A, Terryberry-Spohr L, Wilson KR. Prolonged recovery of memory functioning after mild traumatic brain injury in adolescent athletes. Journal of neurosurgery. 2008;108(3):511-6.
62. Caze T, Vásquez D, Moffatt K, Waple K, Hope D. A Prospective Pilot Study of Anxiety Sensitivity and Adolescent Sports-Related Concussion. Archives of clinical neuropsychology : the official journal of the National Academy of Neuropsychologists. 2021;36(6):930-9.
63. Williams RM, Johnson RS, Snyder Valier AR, Curtis Bay R, Valovich McLeod TC. Evaluating Multiple Domains of Health in High School Athletes With Sport-Related Concussion. Journal of sport rehabilitation. 2021;30(4):545-51.
64. Laufer O, Geva A, Ellis JD, Barber Foss K, Ettinger M, Stern Y, et al. Prospective longitudinal investigation shows correlation of event-related potential to mild traumatic brain injury in adolescents. Informa UK Limited; 2020. p. 871.
65. McGuine TA, Pfaller A, Hetzel S, Broglio SP, Hammer E. A Prospective Study of Concussions and Health Outcomes in High School Football Players. Journal of athletic training. 2020;55(10):1013-9.
66. Broglio SP, Macciocchi SN, Ferrara MS. Sensitivity of the concussion assessment battery. Neurosurgery. 2007;60(6):1050-8.

67. Zuckerman SL, Lee YM, Odom MJ, Solomon GS, Forbes JA, Sills AK. Recovery from sports-related concussion: Days to return to neurocognitive baseline in adolescents versus young adults. Surgical neurology international. 2012;3(1):130.
68. Takagi M, Hearps SJC, Babl FE, Anderson N, Bressan S, Clarke C, et al. Does a computerized neuropsychological test predict prolonged recovery in concussed children presenting to the ED? : Informa UK Limited; 2019. p. 54.
69. Lawrence JB, Haider MN, Leddy JJ, Hinds A, Miecznikowski JC, Willer BS. The King-Devick test in an outpatient concussion clinic: Assessing the diagnostic and prognostic value of a vision test in conjunction with exercise testing among acutely concussed adolescents. Journal of the neurological sciences. 2019;398:91-7.
70. Murdaugh DL, King TZ, Sun B, Jones RA, Ono KE, Reisner A, et al. Longitudinal Changes in Resting State Connectivity and White Matter Integrity in Adolescents With Sports-Related Concussion. Journal of the International Neuropsychological Society. 2018;24(8):781-92.
71. Ellis MJ, Cordingley D, Vis S, Reimer K, Leiter J, Russell K. Vestibulo-ocular dysfunction in pediatric sports-related concussion. Journal of Neurosurgery Publishing Group (JNSPG); 2015. p. 248.
72. Howell DR, Osternig LR, Chou L-S. Adolescents Demonstrate Greater Gait Balance Control Deficits After Concussion Than Young Adults. The American journal of sports medicine. 2015;43(3):625-32.
73. Howell DR, Osternig LR, Koester MC, Chou L-S. The effect of cognitive task complexity on gait stability in adolescents following concussion. Experimental brain research. 2014;232(6):1773-82.
74. Tjarks BJ, Dorman JC, Valentine VD, Munce TA, Thompson PA, Kindt SL, et al. Comparison and utility of King-Devick and ImPACT® composite scores in adolescent concussion patients. Journal of the neurological sciences. 2013;334(1):148-53.
75. Chrisman SP, Rivara FP, Schiff MA, Zhou C, Comstock RD. Risk factors for concussive symptoms 1 week or longer in high school athletes. Brain injury. 2013;27(1):1-9.
76. Maugans TA, Farley C, Altaye M, Leach J, Cecil KM. Resources How To Journal List Pediatrics PMC3255471 Pediatrics. 2012. p. 28.
77. Keightley ML, Yule A, Garland K, Reed N, McAuliffe J, Garton J, et al. Sports-related mild traumatic brain injury in female youths. Unusual presentation of more common disease/injury. 2010;2010(oct29 1):bcr0920092309.
78. Covassin T, Elbin RJ, Nakayama Y. Tracking Neurocognitive Performance following Concussion in High School Athletes. The Physician and sportsmedicine. 2010;38(4):87-93.
79. Lovell MR, Collins MW, Iverson GL, Field M, Maroon JC, Cantu R, et al. Recovery from mild concussion in high school athletes. Journal of neurosurgery. 2003;98(2):296-301.
80. Wu T, Merkley TL, Wilde EA, Barnes A, Li X, Chu ZD, et al. A preliminary report of cerebral white matter microstructural changes associated with adolescent sports concussion acutely and subacutely using diffusion tensor imaging. Brain imaging and behavior. 2017;12(4):962-73.
81. Lau BC, Collins MW, Lovell MR. Sensitivity and Specificity of Subacute Computerized Neurocognitive Testing and Symptom Evaluation in Predicting Outcomes After Sports-Related Concussion. The American journal of sports medicine. 2011;39(6):1209-16.
82. Leddy J, Hinds A, Miecznikowski J, Darling S, Matuszak J, Baker J, et al. Safety and Prognostic Utility of Provocative Exercise Testing in Acutely Concussed Adolescents: A Randomized Trial. Clinical journal of sport medicine. 2018;28(1):13-20.
83. Stephens J, Denckla M, McCambridge T, Slomine B, Mahone E, Suskauer S. Preliminary Use of the Physical and Neurological Examination of Subtle Signs for Detecting Subtle Motor Signs in Adolescents With Sport-Related Concussion. American journal of physical medicine & rehabilitation. 2018;97(6):456-60.
84. Ritchie EV, Emery C, Debert CT. Analysis of serum cortisol to predict recovery in paediatric sport-related concussion. Informa UK Limited; 2018. p. 523.
85. Orr R, Bogg T, Fyffe A, Lam L, Browne G. Graded Exercise Testing Predicts Recovery Trajectory of Concussion in Children and Adolescents. Clinical journal of sport medicine. 2021;31(1):23-30.
86. Haider MN, Worts PR, Viera KB, Villarrubia B, Wilber CG, Willer BS, et al. Postexercise Slowing on the King-Devick Test and Longer Recovery From Sport-Related Concussion in Adolescents: A Validation Study. Journal of Athletic Training/NATA; 2020. p. 482.
87. Elbin R, Sufrinko A, Anderson M, Mohler S, Schatz P, Covassin T, et al. Prospective Changes in Vestibular and Ocular Motor Impairment After Concussion. Journal of neurologic physical therapy. 2018;42(3):142-8.
88. Valier ARPATC, Welch Bacon CEPATC, Bay RCP, Houston MNPATC, Valovich McLeod TCPATC. Validity of Single-Item Patient-Rated Outcomes in Adolescent Football Athletes With Concussion. Archives of physical medicine and rehabilitation. 2016;97(7):1202-5.

89. Dise-Lewis JE, Forster JE, McAvoy K, Stearns-Yoder KA, Bahraini NH, Laker SR, et al. The Natural History of Postconcussion Recovery Among High School Athletes. The journal of head trauma rehabilitation. 2019;34(5):E36-E44.
90. Nolin P, Stipanicic A, Henry M, Joyal CC, Allain P. Virtual reality as a screening tool for sports concussion in adolescents. Brain injury. 2012;26(13-14):1564-73.
91. Howell DR, Wilson JC, Brilliant AN, Gardner AJ, Iverson GL, Meehan WP. Objective clinical tests of dual-task dynamic postural control in youth athletes with concussion. Journal of science and medicine in sport. 2019;22(5):521-5.
92. Purcell L, Harvey J, Seabrook JA. Patterns of Recovery Following Sport-Related Concussion in Children and Adolescents. Clin Pediatr (Phila). 2016;55(5):452-8.
93. Elbin RJ, Sufrinko A, Schatz P, French J, Henry L, Burkhart S, et al. Removal from play after concussion and recovery time. Pediatrics (Evanston). 2016;138(3):1.
94. Lovell MR, Collins MW, Iverson GL, Johnston KM, Bradley JP. Grade 1 or "Ding" Concussions in High School Athletes. The American journal of sports medicine. 2004;32(1):47-54.
95. Kelty-Stephen DG, Qureshi Ahmad M, Stirling L. Use of a Tracing Task to Assess Visuomotor Performance for Evidence of Concussion and Recuperation. Psychological assessment. 2015;27(4):1379-87.
96. Howell DRMS, Osternig LRP, Chou L-SP. Dual-Task Effect on Gait Balance Control in Adolescents With Concussion. Archives of physical medicine and rehabilitation. 2013;94(8):1513-20.
97. Kriz PK, Mannix R, Taylor AM, Ruggieri D, Meehan WP. Neurocognitive Deficits of Concussed Adolescent Athletes at Self-reported Symptom Resolution in the Zurich Guidelines Era. Orthopaedic journal of sports medicine. 2017;5(11):2325967117737307.
98. Howell D, Osternig L, Van Donkelaar P, Mayr U, Chou L-S. Effects of Concussion on Attention and Executive Function in Adolescents. Medicine and science in sports and exercise. 2013;45(6):1030-7.
99. Manning KY, Schranz A, Bartha R, Dekaban GA, Barreira C, Brown A, et al. Multiparametric MRI changes persist beyond recovery in concussed adolescent hockey players. Neurology. 2017;89(21):2157-66.
100. Green SL, Keightley ML, Lobaugh NJ, Dawson DR, Mihailidis A. Changes in working memory performance in youth following concussion. Brain injury. 2018;32(2):182-90.
101. Kraus N, Thompson EC, Krizman J, Cook K, White-Schwoch T, Labella CR. Auditory biological marker of concussion in children. Springer Science and Business Media LLC; 2016.
102. Kontos AP, Elbin RJ, Lau B, Simensky S, Freund B, French J, et al. Posttraumatic Migraine as a Predictor of Recovery and Cognitive Impairment After Sport-Related Concussion. The American journal of sports medicine. 2013;41(7):1497-504.
