# Supplementary material for: Outcome measures used in adolescent sport-related concussion research: a scoping review
Source: BMJ Open. 2024 Sep 10;14(9):e075590. doi: 10.1136/bmjopen-2023-075590 (PMC11409241; doi:10.1136/bmjopen-2023-075590)
Supplement: online supplemental file 2 [file bmjopen-14-9-s002.pdf]

Appendix 2

Outcome measures (n=46)							
**Exercise/Movement/ Balance Testing**	**General Symptoms**	**Imaging**	**Ocular**	**Cognition**	**Mixed Method**	**Other**	**Anxiety/Depression**
BESS/mBESS (n=9)	PCSS (n=42)	MRI (n=14)	VOMS (n=6)	SAC (n=3)	SCAT3 (n=4)	Health and Behaviour Inventory (n=3)	GAD7 (n=1)
BCTT (n=5)	Concussion Symptom Inventory (n=2)	EEG (n=7)	King-Devick (n=3)	Attention Network Test (n=3)	ImpACT (n=21)	Sleep Disturbance Questionnaire (n=1)	AS Index 3 (n=1)
Single Task Tandem Gait (n=1)	Global rating of change scale (n=1)	ECG (n=1)	Unspecified Quantified Ocular Assessment (n=7)	Unspecified Neuropsychological Test Battery (n=1)	ANAM (n=3)	Multidimensional Fatigue Scale (n=1)	PHQ-9 (n=2)
Dual Task Tandem Gait (n=3)			3-D Multiple Object Tracking (n=1)	Verbal and Non-verbal WM task (n=1)	SCAT2 (n=3)	Virtual Reality (n=1)	Satisfaction with life scale (n=1)
Postural Stress Test (n=1)				Task-switching test (n=1)	PedsQOL4 (n=1)	Sleep Accelerometer (n=1)	
Unspecified Static Balance Assessment (n=1)				CogState/Sport (n=4)	PROMIS Paediatric 25 (n=1)	Biomarkers (n=4)	
Unspecified Graded Exercise Test (n=3)					SCAT5 (n=1)	Headache Impact Test 6 (n=2)	
PANESS (n=1)							

Paediatric Clinical Test of Sensory Interaction for Balance (n=1)							
Motion Gait Analysis (n=2)							
Balance Subtest of Bruininks Oseretsky Test of Motor Proficiency (n=1)							
Isometric Handgrip Exercise (n=1)							

BESS/mBESS – Balance Error Scoring System/modified Balance Error Scoring System
BCTT- Buffalo Concussion Treadmill Test
PANESS- Physical and Neurological Examination of Subtle Signs
PCSS- Post-Concussion Symptom Scale
MRI- Magnetic Resonance Imaging
EEG- Electroencephalogram
ECG- Electrocardiogram
VOMS- Vestibular Ocular Motor Screening
SAC-Standardised Assessment of Concussion
SCAT2/3/5- Sport Concussion Assessment Tool 2nd/3rd/5th Edition
ImPACT- Immediate Post-Concussion Assessment and Cognitive Testing
ANAM- Automated Neuropsychological Assessment Metrics
PedsQOL4- Paediatric Quality of Life Inventory 4
PROMIS Paediatric 25- Patient Reported Outcomes Measurement System
GAD7- General Anxiety Disorder 7
PHQ-9- Patient Health Questionnaire 9
AS Index 3- Anxiety Sensitivity Index 3
