# Supplementary material for: Outcome measures used in adolescent sport-related concussion research: a scoping review
Source: BMJ Open. 2024 Sep 10;14(9):e075590. doi: 10.1136/bmjopen-2023-075590 (PMC11409241; doi:10.1136/bmjopen-2023-075590)
Supplement: Supplementary file 3 [file bmjopen-2023-075590.reviewer_comments.pdf]

PEER REVIEW HISTORY

BMJ Open publishes all reviews undertaken for accepted manuscripts. Reviewers are asked to complete a checklist review form (**http://bmjopen.bmj.com/site/about/resources/checklist.pdf**) and are provided with free text boxes to elaborate on their assessment. These free text comments are reproduced below.

ARTICLE DETAILS

**TITLE (PROVISIONAL)**	Outcome measures used in adolescent sport-related concussion research: A Scoping review.
**AUTHORS**	McKee, Connor; Matthews, Mark; Rankin, Alan; Bleakley, Chris

VERSION 1 – REVIEW

**REVIEWER**	McPherson, Jacob<br>University at Buffalo
**REVIEW RETURNED**	20-Sep-2023

**GENERAL COMMENTS**	Thank you for the opportunity to review this manuscript. The study sought to describe outcome measures used in adolescents after sports related concussion. Further, the study attempted to categorize measures by clinical domain and also to describe feasibility of items.<br><br>The stated intent of this work is valuable. A better understanding of commonly used outcome measures, organized by impairments or domains they assess would be a useful addition to the adolescent concussion literature. Informing providers how to select low-cost and low-burden measures to assess individual symptoms or constructs would be of great clinical utility.<br><br>This topic deserves attention but there are some concerns related to the information conveyed within the manuscript. I recommend that revisions are warranted. Please see below for more specific comments.<br><br>1. I am unclear on the definition of “outcome measure” as it is applied to this paper. The manuscript seems to use “assessment tools,” “outcome tools,” “outcome assessment,” and “outcomes” interchangeably. Clinical outcome measures are typically used to measure how a specific construct (e.g., symptom, element of function, behavior) either changes over time or responds to an intervention. Many of the referenced measures (e.g., diffusion tensor imaging, PHQ-9, SCAT, biomarkers, etc.) are used primarily for diagnosing concussion or co-morbid conditions, or aiding in prognosis and are not repeated to measure change over time. It would be preferable to better clarify the difference between an outcome measure and other types of dependent variables. It could be argued that some of the offered “outcome measures” are not clinical outcome measures but more diagnostic tests or assessments. The authors reference the 2014 paper by Collins et al. which offers 6 potential clinical trajectories. Perhaps the definition of outcome measures could be tied to tools that help to determine which of these trajectories a patient’s presentation may align to.

	2. The authors should consider sharing all 59 measures along with the number of studies employing them. Without further description of why a rank order the top 6 measures was provided, it seems as though this was arbitrarily done. Sharing this information could be done using a table included as a supplementary file.<br><br>3. Manuscript page 4, line 25. The SCAT5 is one recommended tool for acute sport concussion assessment. To state that initial assessment of concussion (in general) is based around the SCAT is inaccurate. Further the SCAT begins to lose its diagnostic utility within 2-5 days following injury likely making it a poor “outcome measure”.<br><br>4. Manuscript page 5, line 19. The eligibility criteria appear to be lacking. How did the authors ensure that an appropriate outcome measure was used (doing this would be a challenge without a definition of outcome measure)? Was there consideration of whether measures used would differ based on the assessment of acute vs. chronic injury? It would be useful to break this up into “inclusion criteria” and “exclusion criteria” with relevant factors that are elsewhere mentioned included.<br><br>5. Manuscript page 6, line 23. The term “biomarker” is introduced here for the first time. This should be defined as it appears to mean something different from an outcome measure or clinical domain.<br><br>6. Manuscript page 6, lines 30-36. “Assessment of all 6 clinical profiles was evident…”. These clinical trajectories are not “assessed for”, but rather a clinician performs an examination and evaluates the findings to determine if a patient’s presentation aligns with one of these trajectories or patterns. I believe that the authors instead determined that across the adolescent literature there exists tools to help clinicians elicit findings consistent with these trajectories. Of note, the manuscript from which the 6 clinical trajectories were pulled from (Collins et al, 2014) does not include the term “profile” anywhere in the manuscript. If the authors are embracing this systematic organizational approach they may consider changing the title of this section heading.<br><br>7. Manuscript page 6, lines 47-57. Follow-up assessment is a useful consideration related to outcome measures. It would be helpful to know which outcome measures were most likely to be used during a follow assessment. Where these different from the 6 most commonly used measures offered earlier in the manuscript? Why are some used commonly but not repeated?<br><br>8. Manuscript page 7, lines 19-24. This is an example of why a definition of “outcome measure” offered early in the manuscript would be useful. The listed studies (MRI, EEG, GFAP, etc.) are not clinical outcome measures. They are more closely aligned with diagnostic testing modalities.<br><br>9. Manuscript page 7, lines 34-35. The PCSS is included within the ImPACT. That is unclear based on the current language and the 44% statistic may be misleading as a result.<br><br>10. There is no mention of the ImPACT being utilized within organized sports for baseline testing and subsequent re-testing

	after injury. The “clear preference” (page 7, line 45) likely has very little to do with clinician preference or clinical management but more to do with administrative decision making related to organized sports.<br><br>11. Manuscript page 8, line 12. States that “measures of anxiety/mood and migraine were less well represented in the current adolescent literature” with the PCSS being referenced as one of the most commonly used assessment tools for these domains. At page 7 lines 36-38 the authors state that “anxiety/mood and post-traumatic migraine were the least assessed domains in the current literature” even though 44% of studies employed the PCSS. Is the PCSS a reasonable outcome measure for these domains?<br><br>12. Page 8, line 21. Is the term “outcome assessments” here meant to mean “discharge assessments”? If not, and it means that the time most individuals were evaluated for concussion was between 3 and 21 days after injury, then the 2023 review describing that 80% studies reported a return to sport time of 21 days is irrelevant.<br><br>13. Manuscript page 8, line 46. I was not immediately able to view the full text of the Baker et al. article referenced here but this statement may be more technically accurate if phrased “There is also concern that females may be at greater risk for protracted recovery, and on average….”<br><br>14. Page 8, line 58. I don’t believe that athletes present with impairments of clinical profiles. Instead, their impairments align with one or more clinical profiles. See comment #6 above.<br><br>15. Beginning at page 8, line 59 and extending onto page 9. “Full resolution of all post-concussion-related symptoms” is not required for return to school as the manuscript states. Authors may consider referencing the newer 6th Consensus Statement which explicitly states students may “Gradually progress in school activities until a full day can be tolerated without more than mild* symptom exacerbation.”<br><br>16. Page 9, Conclusion. Is DTI really being used as an outcome measure? Was cognitive/fatigue the most frequently assessed clinical profile because that is what clinicians prefer or at least felt was necessary, or was it most commonly assessed because administrative protocols rely on ImPACT testing for baseline/post-injury testing? I’m also not sure that requiring a “qualified person” makes a tool not feasible.<br><br>17. Page 9, line 35. Do the authors mean to state that the durations of ALL studies included were not long enough to quantify deficits? If some were deemed to include adequate durations, this sentence may need to be updated.<br><br>In summary, this is a valuable topic and the authors have laid out a reasonable approach answering their research questions. With edits, clarifications, and additional descriptions, this work has potential to be a positive addition to the current body of literature.

**REVIEWER**	Kurowski, Brad G.<br>Cincinnati Children's Hospital Medical Center

REVIEW RETURNED	16-Oct-2023

GENERAL COMMENTS	Thank you for the opportunity to review this manuscript. The purpose of this manuscript was to provide<br>a scoping review of outcomes used after sports related concussion (SRC) in adolescents. The authors<br>categorize by clinical domains assessed, follow-up duration, and feasibility of use in an amateur sporting<br>environment. Three electronic databases (Medline, Embase, and CINAHL) were used. 75 articles met<br>inclusion criteria. The main findings were that ImPACT and PCSS were the most common outcomes<br>tracked, with most outcomes measuring aspects of sensorimotor function, including balance,<br>oculomotor, and cognitive domains. Mental health domains, such as anxiety, depression, and other<br>mood disorders were not regularly assessed. Additionally, migraines were not followed regularly. Overall,<br>this article summarizes the state of science. It would be beneficial for the authors to consider expanding<br>more on physiologic and biomarker measurements being used. Also, it would be helpful for the authors<br>to include some discussion of how pre-injury factors might be important in outcome measurement.<br>Further, some recommendations on best measures to use in clinical care based on the state-of-thescience would be helpful, in addition to recommendations for research directions in this area. Note this<br>review was published a priori and follows the PRISMA-ScR checklist.<br>Title: Appropriate.<br>Background: Background overall appropriate. Would consider integrating the main points from the<br>following papers into the background and/or discussion.<br>https://pubmed.ncbi.nlm.nih.gov/29033888/<br>and https://pubmed.ncbi.nlm.nih.gov/28387557/<br>Methods:<br>Seems to be an overall appropriate search. However, I do wonder if any important articles may have<br>been identified if PubMed and/or Euro PMC were also used.<br>Please clarify if “prospective designs” could also include randomized clinical trials.<br>Please clarify how studies with mixed sports and non-sports populations were handled regarding<br>inclusion or exclusion.<br>Results:<br>Good summary of the wide array of domains measured. Feasibility and cost discussion is a nice addition.<br>Discussion:<br>I wonder if there could be more integration and discussion about where there are overlapping domains.<br>I wonder if the authors could make a comment on which outcome measures might be better or more<br>impactful to use and in which setting. For example, which measures are better for pre-season baseline,<br>on the field assessment, and follow-up evaluations.<br>\Imaging and biomarker domains are mentioned, but there seems to be very little discussion of the<br>utility of these outcome measures.<br>Consider following paper on sleep and concussion:<br>https://www.sciencedirect.com/science/article/pii/S0003999318300728?casa_token=bYTxfX9NCJgAAAA

	A:UyL3lAekusP9Ic1C87snaYeq2fmu1LhFbQS9EJzIR0X3fPTMtsQeP8UczsFrq3J8Dc_DvDA1CQ<br>Could authors comment on the use of outcomes without a baseline evaluation?<br>It could be helpful for the authors to state, based on the review, which outcomes seem to have the most<br>clinical utility in which settings. Also, what do authors recommend as the next critical step in research in<br>this area.<br>Please include in limitations that generalization to non-sports mechanisms of injury is limited based on<br>the focus of this scoping review.<br>Also, could authors comment on whether papers included used standard definition of concussion and/or<br>mild TBI. This might be a limitation if there is variability in how concussion was defined for the studies<br>included. Please consider following references on definition of mTBI:<br>https://pubmed.ncbi.nlm.nih.gov/37211140/ , there may be others to consider as well.<br>Appendix 1: It might be helpful to the reader if the authors could include a grading rating of the strength<br>of the study along with a rating of potential bias in the table.<br>Figures: overall appropriate.

VERSION 1 – AUTHOR RESPONSE

Reviewer 1 Comments

1. I am unclear on the definition of “outcome measure” as it is applied to this paper. The manuscript seems to use “assessment tools,” “outcome tools,” “outcome assessment,” and “outcomes” interchangeably. Clinical outcome measures are typically used to measure how a specific construct (e.g., symptom, element of function, behavior) either changes over time or responds to an intervention. Many of the referenced measures (e.g., diffusion tensor imaging, PHQ-9, SCAT, biomarkers, etc.) are used primarily for diagnosing concussion or co-morbid conditions, or aiding in prognosis and are not repeated to measure change over time. It would be preferable to better clarify the difference between an outcome measure and other types of dependent variables. It could be argued that some of the offered “outcome measures” are not clinical outcome measures but more diagnostic tests or assessments. The authors reference the 2014 paper by Collins et al. which offers 6 potential clinical trajectories. Perhaps the definition of outcome measures could be tied to tools that help to determine which of these trajectories a patient’s presentation may align to.

   Response: Thank you for your feedback. We have decided to keep the term ‘outcome measure’ however we have included how this was defined. See line 133-138. *“An outcome measure was any non-demographic outcome variable that was assessed at least once post-concussion, they could be either subjective (PROMs) or objective (imaging, biomarker, physical performance test).”*

2. The authors should consider sharing all 59 measures along with the number of studies employing them. Without further description of why a rank order the top 6 measures was provided, it seems as though this was arbitrarily done. Sharing this information could be done using a table included as a supplementary file.

   Response: We have included a table which includes all outcome measures alongside their frequency of use; please see Appendix 2.

3. Manuscript page 4, line 25. The SCAT5 is one recommended tool for acute sport concussion assessment. To state that initial assessment of concussion (in general) is based around the SCAT is inaccurate. Further the SCAT begins to lose its diagnostic utility within 2-5 days following injury likely making it a poor “outcome measure”.

   Response: We have changed the wording of this sentence. It now reads “one widely used assessment battery is the Standardised Concussion Assessment Tool (SCAT5)”. See line 87.

4. Manuscript page 5, line 19. The eligibility criteria appear to be lacking. How did the authors ensure that an appropriate outcome measure was used (doing this would be a challenge without a definition of outcome measure)? Was there consideration of whether measures used would differ based on the assessment of acute vs. chronic injury? It would be useful to break this up into “inclusion criteria” and “exclusion criteria” with relevant factors that are elsewhere mentioned included.

   Response: We didn’t seek to explore whether an appropriate outcome measure was used, rather our purpose was to audit the range of concussion outcome measures currently being used in adolescent sports. We did not differentiate based on the assessment of acute vs. chronic injury. We have now included an extra line in the eligibility criteria section highlighting that studies must have included at least one follow-up time point post-concussion to be included. See line 122-23.

5. Manuscript page 6, line 23. The term “biomarker” is introduced here for the first time. This should be defined as it appears to mean something different from an outcome measure or clinical domain.

   Response: Certain studies which were found in the review included the use of biomarkers to assess concussion recovery. For the purpose of this review, as something which tracked recovery over time, we included biomarkers as outcome measures. *SRC outcome measures using imaging or biomarkers were also represented, and included Magnetic Resonance Imaging, Electrocardiogram, Electroencephalogram, glial and neuronal blood biomarkers.* See line 176-179.

6. Manuscript page 6, lines 30-36. “Assessment of all 6 clinical profiles was evident…”. These clinical trajectories are not “assessed for”, but rather a clinician performs an examination and evaluates the findings to determine if a patient’s presentation aligns with one of these trajectories or patterns. I believe that the authors instead determined that across the adolescent literature there exists tools to help clinicians elicit findings consistent with these trajectories. Of note, the manuscript from which the 6 clinical trajectories were pulled from (Collins et al, 2014) does not include the term “profile” anywhere in the manuscript. If the authors are embracing this systematic organizational approach they may consider changing the title of this section heading.

   Response: Thank you for this feedback. We have addressed this comment and updated the wording of these sections accordingly. We have updated the document throughout to include the term “construct” rather than “clinical profile”. This term is used frequently in the COSMIN reporting guidelines for studies on measurement properties of patient reported outcome measures. https://www.cosmin.nl/wp-content/uploads/COSMIN-reporting-guideline_1.pdf

7. Manuscript page 6, lines 47-57. Follow-up assessment is a useful consideration related to outcome measures. It would be helpful to know which outcome measures were most likely to be used during a follow assessment. Where these different from the 6 most commonly used measures offered earlier in the manuscript? Why are some used commonly but not repeated?

   Response: We have included a sentence in the ‘Outcome Measures’ section highlighting that these methods were most likely to be used throughout all stages of tracking recovery and the reason for this, see line 165-166.

8. Manuscript page 7, lines 19-24. This is an example of why a definition of “outcome measure” offered early in the manuscript would be useful. The listed studies (MRI, EEG, GFAP, etc.) are not clinical outcome measures. They are more closely aligned with diagnostic testing modalities.

   Response: We have now defined the term outcome measure (see line 133-137). “*An outcome measure was any non-demographic outcome variable that was assessed at least once post-concussion, they could be either subjective (PROMs) or objective (imaging, biomarker, physical performance test)”*

9. Manuscript page 7, lines 34-35. The PCSS is included within the ImPACT. That is unclear based on the current language and the 44% statistic may be misleading as a result.

   Response: Thanks for highlighting this important point. We have updated the article and made amendments to tables and figures to reflect the PCSS’s inclusion in ImPACT, and number of times used in studies. See also line 222-223.

10. There is no mention of the ImPACT being utilized within organized sports for baseline testing and subsequent re-testing after injury. The “clear preference” (page 7, line 45) likely has very little to do with clinician preference or clinical management but more to do with administrative decision making related to organized sports.

   Response: We have removed the ‘clear preference’ and replaced it with: *“The ImPACT and PCSS outcome measures were the most frequently utilised in this study, and this is likely due to their ease of use, the wide variety of areas they assess and relative low cost.”* See line 229-31.

11. Manuscript page 8, line 12. States that “measures of anxiety/mood and migraine were less well represented in the current adolescent literature” with the PCSS being referenced as one of the most commonly used assessment tools for these domains. At page 7 lines 36-38 the authors state that “anxiety/mood and post-traumatic migraine were the least assessed domains in the current literature” even though 44% of studies employed the PCSS. Is the PCSS a reasonable outcome measure for these domains?

   Response: We have included a line to address in the discussion. *“Although the PCSS assesses for anxiety in its questions, it fails to provide a score for anxiety separately which limits its usefulness in assessing this domain.”* See line 252-54.

12. Page 8, line 21. Is the term “outcome assessments” here meant to mean “discharge assessments”? If not, and it means that the time most individuals were evaluated for concussion was between 3 and 21 days after injury, then the 2023 review describing that 80% studies reported a return to sport time of 21 days is irrelevant.

   Response: We have addressed this to improve clarity. It is meant to explain that most follow-up assessments after a concussion occur within 3-weeks (not necessarily discharge). Although this period maybe be long-enough for most to recover (mainly adults), a subset of younger athletes experience a protracted recovery trajectory from concussion and require longer follow-up times in research to fully understand the deficits associated with concussion in this population. See line 261-272.

13. Manuscript page 8, line 46. I was not immediately able to view the full text of the Baker et al. article referenced here but this statement may be more technically accurate if phrased “There is also concern that females may be at greater risk for protracted recovery, and on average….”

   Response: Thank you, we have revised this sentence based on your feedback, see line 281-83.

14. Page 8, line 58. I don't believe that athletes present with impairments of clinical profiles. Instead, their impairments align with one or more clinical profiles. See comment #6 above.

    Response: We have taken this on board and revised our wording to reflect your feedback. Please see our response to point 6 above..

15. Beginning at page 8, line 59 and extending onto page 9. "Full resolution of all post-concussion-related symptoms" is not required for return to school as the manuscript states. Authors may consider referencing the newer 6th Consensus Statement which explicitly states students may "Gradually progress in school activities until a full day can be tolerated without more than mild* symptom exacerbation."

    Response: Thank you, we have revised this sentence to imply a return to competitive sports. Please see line 294.

16. Page 9, Conclusion. Is DTI really being used as an outcome measure? Was cognitive/fatigue the most frequently assessed clinical profile because that is what clinicians prefer or at least felt was necessary, or was it most commonly assessed because administrative protocols rely on ImPACT testing for baseline/post-injury testing? I'm also not sure that requiring a "qualified person" makes a tool not feasible.

    Response: We have revised our conclusion to reflect the findings from our study; being that ImPACT and PCSS were the two most commonly used outcome measures. We have revised our wording around feasibility of using these outcome measures to include associated costs and requirements of highly qualified persons such as medical professionals, which may not be readily accessible in most adolescent sports.

17. Page 9, line 35. Do the authors mean to state that the durations of ALL studies included were not long enough to quantify deficits? If some were deemed to include adequate durations, this sentence may need to be updated.

    Response: This sentence has been updated to reflect future research needs to consider longer follow-up periods to explore and quantify possible persistent deficits. Please see line 323-326.

Reviewer 2 Comments

1. **Title**- Appropriate.
2. **Background**- Overall appropriate. Would consider integrating the main points from the following papers into the background and/or discussion. https://pubmed.ncbi.nlm.nih.gov/29033888/ and https://pubmed.ncbi.nlm.nih.gov/28387557/
   Response: Thank you for these suggestions, I have now referenced the first paper in the background.

3. **Methods**- Seems to be an overall appropriate search. However I do wonder if any important articles may have been identified if PubMed and/or Euro PMC were also used.
   Response: It is possible that more papers may have been found by including more databases, however with the large number of papers (21102) we identified in this review we believe that we have provided a sufficient synthesis of the current research.

4. Please clarify if "prospective designs" could also include randomised clinical trials.
   Response: Prospective designs in this study may have included randomised clinical trials, provided that they included one or more follow-up time post-concussion.

5. Please clarify how studies with mixed sports and non-sports populations were handled regarding inclusion or exclusion.

Response: This study aimed to include only those who were involved with sports. However 5 studies included a small sample of non-sport players alongside sport players and these were also included. This detail is mentioned now on line 152-154.

6. **Results-** Good summary of the wide array of domains assessed. Feasibility and cost discussion is a nice addition.
7. **Discussion-** I wonder if there could be more integration and discussion about where there are overlapping domains.
   Response: We mention in the discussion that SRC is heterogenous and requires multi-modal assessment (line 227-228) to combat this.

8. I wonder if the authors could make a comment about which outcome measures might be better or more impactful to use and in which setting. For example, which measures are better for pre-season baseline, on the field assessment, and follow-up evaluations.
   Response: Thank you for this feedback. This is an excellent point, however given the scope of this review we do not feel best placed to provide an answer to this question. We believe that this would require further investigation beyond this review.

9. Imaging and biomarker domains are mentioned, but there seems to be very little discussion of the utility of these outcome measures.
   Response: We have now mentioned that these outcomes may be less feasible for use in an adolescent sporting environment. See line 209-213.

10. Consider the following paper on sleep and concussion: https://www.sciencedirect.com/science/article/pii/S0003999318300728?casa_token=bYTxfX9NCJgAAAAA:UyL3lAekusP9Ic1C87snaYeq2fmu1LhFbQS9EJzIR0X3fPTMtsQeP8UczsFrq3J8Dc_DvDA1CQ
    Response: This is an interesting paper, thank you for sharing it with us. Had this been uncovered as part of the review process, it would have likely been included.

11. Could authors comment on the use of outcomes without a baseline evaluation?
    Response: We can't suggest whether it is better or not to use a baseline evaluation based on this review, however we have added a sentence in the discussion line 245-47, "Therefore it is important to consider pre-existing conditions at baseline, to understand the effects this may have on follow-up assessment."

12. It could be helpful for the authors to state, based on the review, which outcomes seem to have most clinical utility in which settings. Also, what do authors recommend as the next critical step in research in this area.
    Response: The review itself comments on the feasibility of certain assessments, and with no clear gold-standard it would be challenging to recommend specific ones for use in specific settings. Given the lack of studies which extend beyond 6-weeks post-concussion, we have suggested that future concussion research in adolescents should extend beyond the acute stages of recovery to fully quantify the deficits associated with concussion (see line 324-26).

13. Please include in the limitations that generalization to non-sports mechanisms of injury is limited based on the focus of this scoping review.
    Response: added in the limitations section, see line 310-312.

14. Also, could authors comment on whether papers included used standard definition of concussion and/or mild TBI. This might be a limitation if there is variability in how concussion was defined for the studies included. Please consider following references on definition of mTBI: https://pubmed.ncbi.nlm.nih.gov/37211140/
    Response: We have addressed and included this reference in the limitations section. See line 312-13.

15. **Appendix 1:** it might be helpful to the reader if the authors could include a grading rating of the strength of the study along with a rating of potential bias in the table.
Response: Thank you for this comment. We agree that this may be helpful, however we did not conduct this analysis of bias and feel that we would not be best positioned to include this post-analysis. As this was a scoping review it is not standard practise to include an assessment of the risk of bias.
16. **Figures:** Overall appropriate.

New comments from 4/3/24

18. You have indicated a funder/s for your paper. Please ensure to provide an award/grant number for your funder/s in the main document file and in ScholarOne.
Response: See line 347.

19. Figures can be supplied in TIFF, JPG or PDF format (figures in document, excel or powerpoint format will not be accepted), we also request that they have a resolution of at least 300 dpi and 90mm x 90mm of width.
Response: We have updated the format of our figures and tables to be in PDF format.

20. Please re-upload your Supplementary files in PDF format.
Response: We have re-uploaded our files to be in PDF format.

21. Please ensure that your "Corresponding author" and provide a "Corresponding author's email address" in your main document and ScholarOne submission system is the same.
Response: We have ensured that the corresponding author is consistent on both scholar one and in the manuscript.

22. Please provide an Article summary section consisting of the heading: 'Strengths and limitations of this study', and containing up to five (5) and a minimum of three (3) bullet points that relate specifically to the study reported. This should be placed after the abstract.
Response: Now included. See line 54-60.

23. Authors must include a statement in the Methods section of the manuscript under the sub-heading 'Patient and Public Involvement.
Response: We have now included a patient and public involvement statement. See line 148-151.

24. **No ethical approval statement in statements section.**
Response: Now included. See line 350-353.

25. Please provide a "Data availability statement" in your main document file as shown in ScholarOne.
Response: Now included. See line 341-43.

26. Please provide a more detailed contributorship statement. It needs to mention all the names/initials of authors along with their specific contribution/participation for the article.
Response: See line 12-19.

VERSION 2 – REVIEW

**REVIEWER**	McPherson, Jacob<br>University at Buffalo
**REVIEW RETURNED**	02-Apr-2024

**GENERAL COMMENTS**	Thank you for your consideration of recommended revisions.
